# Hypomethylation of GNA15 Promotes Pancreatic Ductal Adenocarcinoma Progression and Macrophage M2 Polarization via STAT3‐CXCL8 Axis

**DOI:** 10.1002/advs.76860

**Published:** 2026-07-30

**Authors:** Weihui Guo, Zhenyuan Qian, Lei Wang, Weilang Xu, Yuxin Weng, Kai Jiang, Zaiyuan Ye, Ji Xu, Guangyuan Song

**Affiliations:** ^1^ Department of General Surgery, Cancer Center, Division of Gastrointestinal and Pancreatic Surgery Zhejiang Provincial People's Hospital, Affiliated People's Hospital Hangzhou Medical College Hangzhou Zhejiang China; ^2^ Graduate School of Zhejiang Chinese Medical University Hangzhou Zhejiang China; ^3^ Department of Radiology Zhejiang Medical & Health Group Quzhou Hospital (Zhejiang Quhua Hospital) Quzhou Zhejiang China; ^4^ Fuyang Branch of Zhejiang Provincial People's Hospital Hangzhou Zhejiang China; ^5^ Department of General Surgery, Cancer center Division of Hepatobiliary and Pancreatic Surgery Zhejiang Provincial People's Hospital Affiliated People's Hospital Hangzhou Medical College Hangzhou Zhejiang China

**Keywords:** chemoresistance, CXCL8, GNA15, macrophage polarization, pancreatic cancer, STAT3

## Abstract

The complexity of pancreatic ductal adenocarcinoma (PDAC) progression, coupled with the lack of effective immunotherapy, underscores the imperative to deepen our understanding of its mechanisms and identify suitable immune‐targeted interventions. The G protein alpha subunit 15 (GNA15) is significantly upregulated in PDAC and correlates with poor prognosis in patients with PDAC. Mechanistically, our study demonstrates that TET3 upregulates GNA15 expression through demethylation in PCs, and the highly expressed GNA15 activates the phosphorylation of STAT3 via the GP130‐JAK signaling pathway, thereby promoting the expression and release of CXCL8. Subsequently, CXCL8 released by PCs binds to CXCR1 or CXCR2 in macrophages to promote M2 polarization. Additionally, high GNA15 expression was associated with Gemcitabine resistance, and the combination of Reparixin and Gemcitabine has shown favorable antitumor efficacy in PDAC. Collectively, we elucidated that GNA15 drives PDAC progression and M2 macrophage polarization via the STAT3‐CXCL8 axis and established targeting GNA15‐STAT3‐CXCL8 as a novel strategy to improve PDAC therapies.

## Graphical abstract

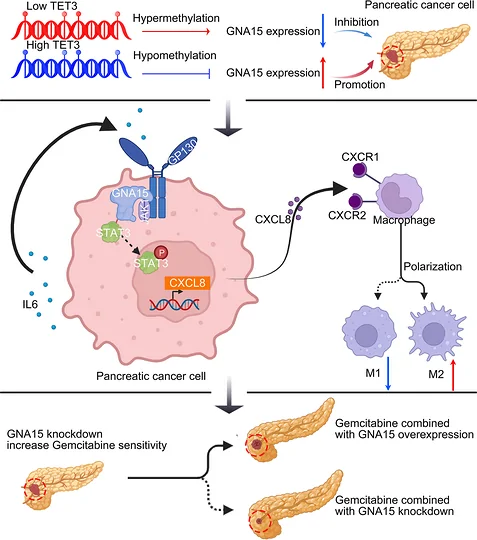

## 1 Introduction

Pancreatic ductal adenocarcinoma (PDAC) is a highly fatal tract malignancy, with a 5‐year survival rate of only 13% for patients [1]. By 2030, PDAC is projected to become the second leading cause of cancer‐related deaths worldwide [2]. Current treatment strategies mainly rely on surgery and chemotherapy [3]. Due to the insidious onset of early symptoms, most patients are diagnosed at an advanced stage, making surgical cure exceedingly difficult [4]. Moreover, chemotherapy regimens based on Gemcitabine have shown limited efficacy [5]. KRAS [6] and TP53 [7] are recognized as the primary driver genes in pancreatic cancer. However, their potential as therapeutic targets is limited by inherent challenges in drug development and targeted intervention [8]. Recently, with the rapid advancement of immunotherapy, it is emerging as a promising avenue for cancer treatment. Unfortunately, its effectiveness in pancreatic cancer remains limited [9]. These underline an urgent need to identify novel therapeutic targets and a deep exploration of the immune responses involved in PDAC.

The pathogenesis and progression of PDAC are highly complex and orchestrated by diverse molecular malignancy [10, 11]. DNA methylation, a pivotal epigenetic driver in cancer, contributes to tumor progression [12, 13]. Our previous work has shown that DNA methylation facilitates malignancy by silencing tumor suppressor genes, such as GNA14, thereby promoting aggressive proliferation, invasion, and metastasis [14, 15]. GNA15 is a member of the G protein alpha subunit family. It has been reported to be overexpressed in PDAC and correlated with poor patient prognosis [16]. However, the function and mechanisms of GNA15 in PDAC have not been elucidated. GNA15 is closely linked to the tumor immune microenvironment [17], and its abnormal expression will influence the secretion of multiple cytokines via STAT3, such as CXCL8 [18]. STAT3 belongs to the STAT family, and its abnormal activation contributes to tumor progression [19]. This study demonstrates that GNA15 drives PDAC progression via activating STAT3‐CXCL8.

Immune cells in the tumor microenvironment (TME) play a key role in cancer initiation and progression [20]. Tumor‐associated macrophages (TAMs) foster the malignant proliferation of tumor cells through the secretion of cytokines such as IL‐10 and TGF‐β [21, 22]. PDAC is commonly characterized by the infiltration of numerous TAMs. It has been reported that substantial TAMs infiltration is closely linked to resistance to Gemcitabine [23]. Therefore, suppressing TAMs infiltration remains an effective strategy to improve its therapeutic efficacy.

In this study, we employed both in vitro and in vivo models to investigate the role of GNA15 in the malignant progression of PDAC. Through RNA sequencing, co‐immunoprecipitation (Co‐IP), and chromatin immunoprecipitation (ChIP) assays, we demonstrated that GNA15 promotes PDAC progression via the STAT3–CXCL8 axis. Finally, we confirmed that the combination of Reparixin and Gemcitabine is an effective therapeutic approach in PDAC.

## 2 Results

### 2.1 GNA15 is Upregulated in PDAC and Associated With a Poor Prognosis

We focus on investigating the role of G proteins in cancer [14]. Recently, GNA15 was reported to be a potential oncogene regulated in PDAC [16]. However, the role of GNA15 in PDAC has not been fully defined. Therefore, we further investigated the clinical value and the function of GNA15 in PDAC. We found that the mRNA level of GNA15 was upregulated in PDAC specimens compared with normal controls in TCGA and GEO databases (GSE62452, GSE28735, GSE16515, GSE211895, and GSE165399) (Figure 1A–D and Figure S1A–L). To further verify these data, we collected 30 pairs of PDAC tissues and normal pancreatic tissues for qRT‐PCR and Western blot. The results revealed that the mRNA and protein levels of GNA15 were upregulated in PDAC tissues relative to normal pancreatic tissues (Figure 1E–G). Subsequently, we downloaded the clinical information of PDAC patients from TCGA to deep analysis. The analysis showed that the high expression of GNA15 was associated with poor overall survival (OS) and progression‐free survival (RFS) (Figure 1H and Figure S1M). The univariate COX analysis indicated that multiple parameters, including GNA15 expression level, clinical N, and M stages, were significantly correlated with the prognosis of patients with PDAC. These parameters were incorporated into the nomogram prediction models. The results of the models revealed that all calibration curves yielded robust predictive efficacy for the 1‑year, 2‑year, and 3‑year clinical outcomes of the three models (Figure 1I–K). In addition, IHC was used to detect GNA15 in 120 PDAC specimens. We observed that the IHC score of GNA15 was related to sex, lymph node metastasis, vascular invasion, tumor size, and prognosis (Figure 1L). Taken together, our integrated multi‐omics analysis suggests that GNA15 is upregulated in PDAC, and this abnormal overexpression is associated with poor prognosis in patients.

**FIGURE 1:**
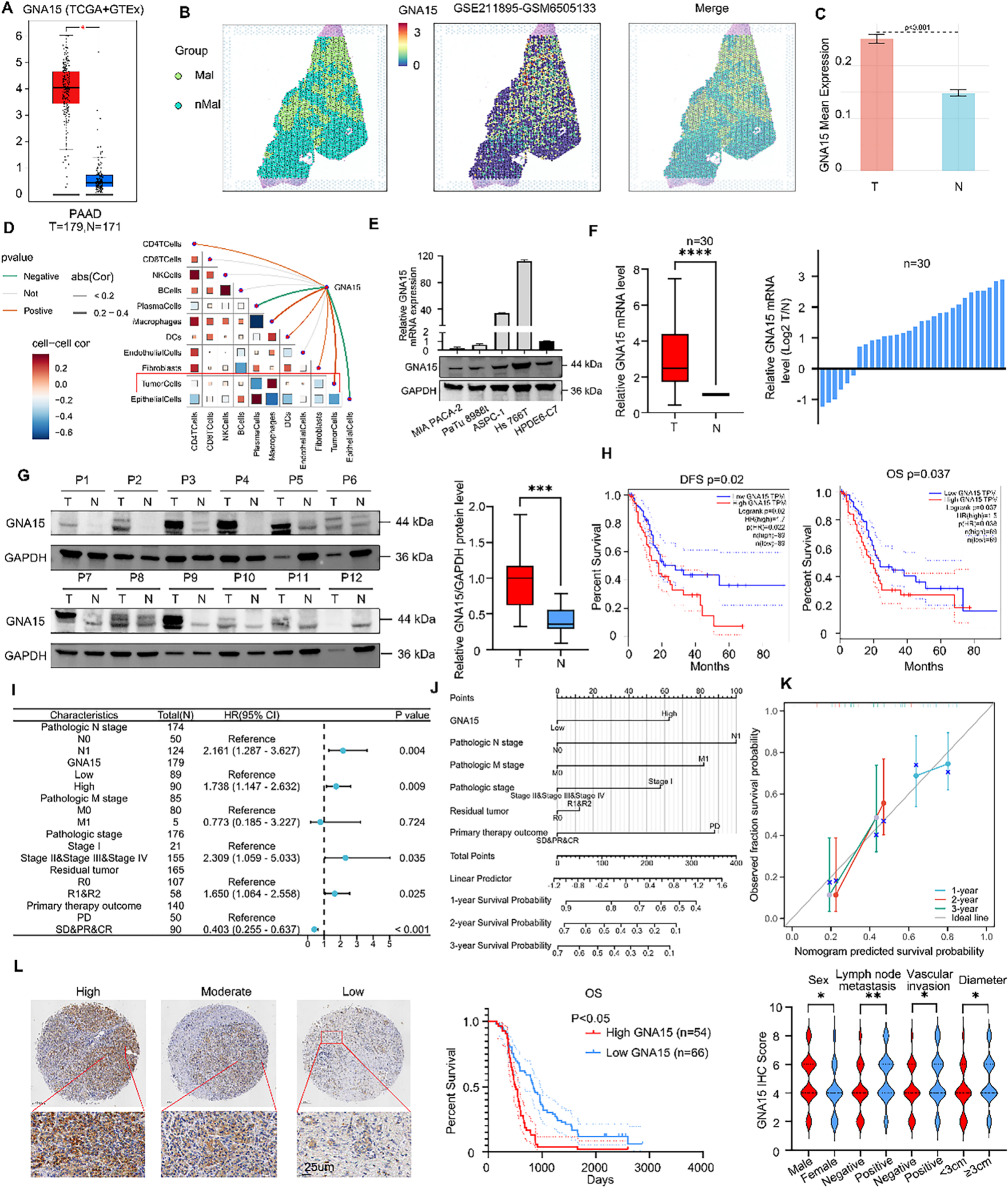
GNA15 is upregulated in PDAC and associated with a poor prognosis. (A) Expression levels of GNA15 mRNA in PDAC tissues and normal controls from GEPIA database. (B) Spatial distribution map of GNA15 in the PDAC spatial transcriptome from GSE211895 dataset. (C) Spatial distribution map and quantitative statistics map of GNA15 in the PDAC spatial transcriptome from GSE211895 dataset. (D) Correlation analysis map between GNA15 and different cells in the PDAC spatial transcriptome from GSE211895 dataset. (E) Expression levels of GNA15 in different PDAC cell lines detected by qRT‐PCR and Western blot. (F) Expression levels of GNA15 mRNA in 30 pairs of PDAC patient specimens and adjacent normal specimens detected by qRT‐PCR. (G) Protein expression levels of GNA15 in 12 pairs of PDAC patient specimens and adjacent normal specimens detected by Western blot. (H) The association of GNA15 mRNA levels with OS and RFS of PDAC patients in the TCGA database. (I) Univariate analysis table related to PDAC from the TCGA database. (J, K) Nomogram and calibration plot related to PDAC from the TCGA database. (L) Representative IHC staining images and clinical correlation analysis of GNA15 expression in 120 PAAD tissues. The *t*‐test was used for comparisons between two groups, and ANOVA was used for multiple group comparisons. *p < 0.05. **p < 0.01. ***p < 0.001. ****p < 0.0001.

### 2.2 GNA15 Drives PDAC Malignant Progression In Vitro and In Vivo

To explore the role of GNA15 in PDAC progression, we stably knockdown GNA15 in ASPC‐1 and Hs 766T, and overexpression GNA15 in PaTu 8988t and MIA PaCa‐2 (Figure 2A,B and Figure S2A,B). The results of CCK‐8 and colony formation assays showed that the proliferation and colony formation capacity of sh‐GNA15 ASPC‐1 and Hs766T cells was significantly inhibited. In contrast, the capacity of proliferation in PaTu 8988t and MIA PaCa‐2 cells with GNA15 overexpression was remarkably enhanced (Figure 2C,D and Figure S2C,D). Transwell assays were used to assess the alteration in the invasive and migratory abilities of PCs induced by GNA15. Silencing of GNA15 suppressed the invasive and migratory abilities of PCs, whereas opposite experimental results were obtained following GNA15 overexpression (Figure 2E and Figure S2E). Subsequently, nude mouse subcutaneous tumor models were established to further explore the in vivo biological function of GNA15 in PDAC. Compared with the NC group, the tumor volume and weight in sh‐GNA15 groups were significantly reduced (Figure 2F). Conversely, GNA15 overexpression signally increased the tumor volumes and weight (Figure 2G). IHC staining demonstrated that GNA15 overexpression upregulated the expression of Ki67, a proliferation marker, in subcutaneous tumors (Figure 2H). In the nude mouse lung metastasis model, depletion of GNA15 suppressed the metastasis of PDAC (Figure 2I). Flow cytometry was used to inspect the effects of GNA15 on cell cycle and apoptosis of PCs. Inhibition of GNA15 arrested PCs at the S phase and induced their apoptosis, whereas the contrary conclusions were observed following GNA15 overexpression (Figure S3A–H). Collectively, these findings illustrate that GNA15 promotes the malignant progression of PDAC, while GNA15 knockdown can effectively suppress the progression of PDAC. This highlights the potential of GNA15 as a therapeutic target.

**FIGURE 2:**
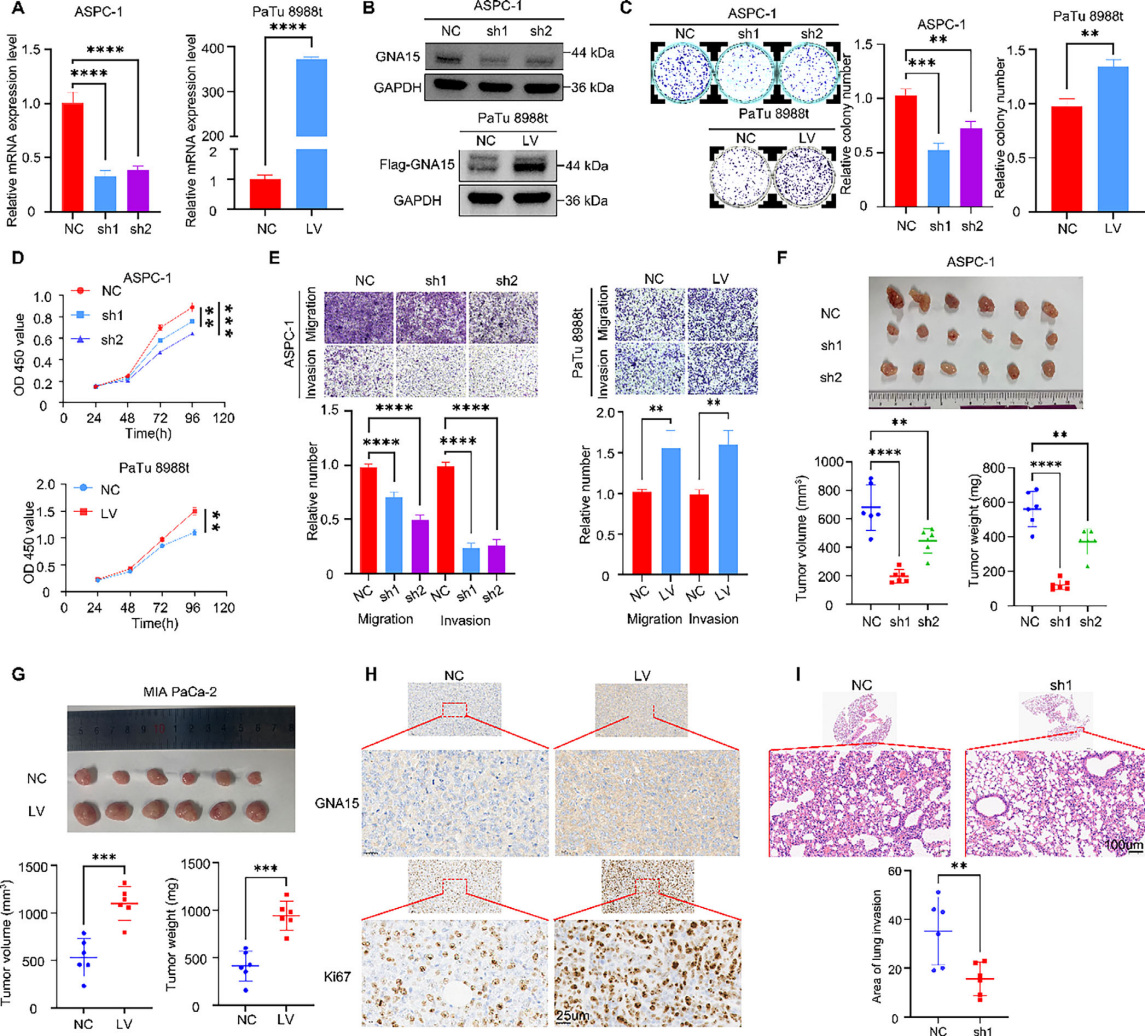
GNA15 drives PDAC malignant progression in vitro and in vivo. (A, B) Verification of lentivirus‐mediated stably sh‐GNA15 ASPC‐1 cells and stably LV‐GNA15 PaTu 8988t cells by qRT‐PCR and Western blot. (C–E) ASPC‐1 and PaTu 8988t cells were subjected to colony formation assay, CCK‐8 assay and Transwell assay. (F) ASPC‐1 cells stably expressing sh‐NC and sh‐GNA15 were transplanted subcutaneously into nude mice, and xenograft tumor assay was performed for detection. (G) MIA PaCa‐2 cells with stable overexpression were transplanted subcutaneously into nude mice, and xenograft tumor assay was performed for detection. (H) Representative IHC staining images of GNA15 and Ki‐67 in tumor tissues of BALB/c nude mice. (I) HE staining images of lung tissues of BALB/c nude mice in the lung metastasis model. The *t*‐test was used for comparisons between two groups, and ANOVA was used for multiple group comparisons. *p < 0.05. **p < 0.01. ***p < 0.001. ****p < 0.0001.

### 2.3 GNA15 Expression Was Regulated by DNA Demethtlase TET3

DNA methylation is an important epigenetic regulatory mechanism and has emerged as a valuable cancer biomarker [24]. The level of DNA methylation is closely associated with gene expression levels, which is primarily regulated by DNA methyltransferases (DNMTs) and ten‐eleven translocation (TETs) demethylases [25, 26]. To investigate the DNA methylation level of GNA15 in PDAC, we performed analyses using SMART (Figure S4A). We found that the DNA methylation level of GNA15 detected by the cg26482939 probe was significantly decreased in tumors (Figure S4B). The methylation level of GNA15 was negatively correlated with its mRNA level (Figure S4C). In addition, the results of further analysis indicated that low methylation level of GNA15 was associated with PDAC metastasis and poor prognosis of patients (Figure S4D–F). Subsequently, we conducted correlation expression analysis in the TCGA‐PDAC dataset, and the results showed that TET3 had the most significant correlation with GNA15 (Figure S4G,H). To determine whether TET3 regulates GNA15 expression, we used two approaches: siRNA‐mediated knockdown and Bobcat339 [27] treatment. Following TET3 knockdown, the expression level of GNA15 was concomitantly decreased (Figure S4I,J). Similarly, GNA15 protein level in PCs was also reduced after Bobcat339 treatment (Figure S4K). Functional experiments demonstrated that Bobcat339 can inhibit the malignant progression of PDAC induced by GNA15 (Figure S4L,M). Finally, the CHIP‐qPCR results indicated that TET3 directly binds to the promoter region of GNA15 (Figure S4N). To summarize, these results demonstrate that the DNA demethylase TET3 is an upstream element regulator of GNA15.

### 2.4 GNA15 Drives PDAC Malignant Progression via CXCL8

To further explore the downstream mechanism of GNA15 in PDAC, ASPC‐1 with NC and sh‐GNA15 were employed for transcriptome sequencing. Subsequent enrichment analysis of the sequencing data revealed that GNA15 was significantly correlated with chemokines CXCL1, CXCL3, and CXCL8 (Figure 3A,B). Data from TCGA and GEO demonstrated a positive relationship between GNA15 and CXCL8 expression, whereas no such correlation was observed between GNA15 and CXCL1/CXCL3 (Figure 3C and Figure S5A–E). These results implicated CXCL8 as a potential downstream target gene of GNA15.To further verify the correlations between GNA15 and these three chemokines, qRT‐PCR was used to detect the mRNA expression levels of CXCL1, CXCL3, and CXCL8. Results indicated that knockdown of GNA15 led to a significant reduction in the mRNA level of CXCL8, however, no obvious changes were observed in the mRNA expression of CXCL1 and CXCL3 (Figure 3D). Inhibition of CXCL8 expression following GNA15 knockdown was observed by IHC staining (Figure 3E). Furthermore, mRNA expression levels of CXCL8 and GNA15 exhibited a significant positive correlation across different clinical cohorts of PDAC patients (Figure 3F,G). CXCL8 was observed to be abnormally overexpressed in PDAC with poor prognosis of patients (Figure 3H and Figure S5F,G). Combined analysis of GNA15 and CXCL8 confirmed that patients with concurrent high expression of both GNA15 and CXCL8 had a worse prognosis compared with those with concurrent low expression of both (Figure 3I).

**FIGURE 3:**
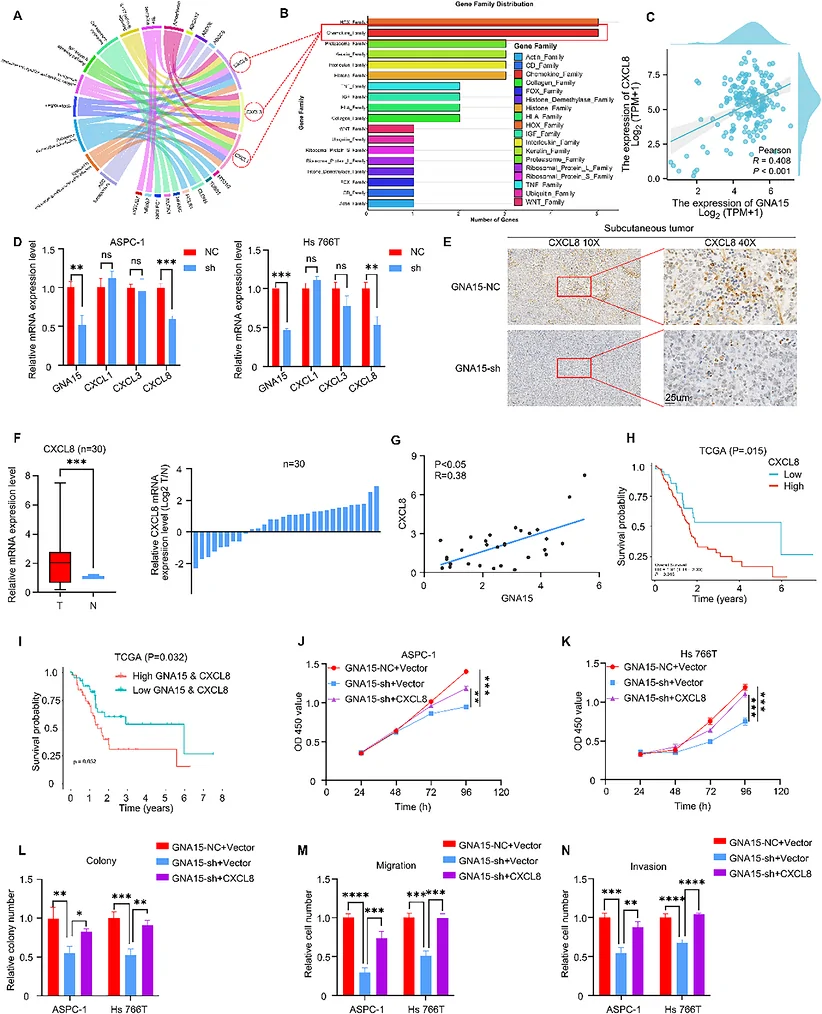
GNA15 drives PDAC malignant progression via CXCL8. (A, B) Changes in the transcription levels of sh‐NC and sh‐GNA15 ASPC‐1 cells were detected by RNA sequencing. R studio was used for enrichment analysis. (C) The association of the mRNA expression levels of GNA15 with CXCL8 in TCGA‐PDAC. (D) ASPC‐1 and Hs 766T cells with knockdown of GNA15 were subjected to qRT‐PCR assays to detect the mRNA levels of CXCL1, CXCL3 and CXCL8. (E) Representative IHC staining images of CXCL8 in tumor tissues of BALB/c nude mice. (F) Expression levels of CXCL8 mRNA in 30 pairs of PDAC patient specimens and adjacent normal specimens detected by qRT‐PCR. G) Relationship map between GNA15 and CXCL8 from qRT‐PCR results. (H) R studio was used to perform a analysis of association between CXCL8 mRNA levels and OS of PDAC patients in the TCGA database. (I) R studio was used to perform a combined analysis of the correlation between GNA15 and CXCL8 mRNA levels with OS of PDAC patients in the TCGA database. (J, K) CCK‐8 assay was performed on PCs expressing sh‐NC, sh‐GNA15, and sh‐GNA15+CXCL8. (L) Colony formation assay was conducted on PCs expressing sh‐NC, sh‐GNA15, and sh‐GNA15+CXCL8. (M, N) Transwell assay was conducted on PCs expressing sh‐NC, sh‐GNA15, and sh‐GNA15+CXCL8. The *t*‐test was used for comparisons between two groups, and ANOVA was used for multiple group comparisons. *p < 0.05. **p < 0.01. ***p < 0.001. ****p < 0.0001.

To investigate whether GNA15 promotes the malignant progression of PDAC by regulating CXCL8 expression, we transfected plasmid myc‐CXCL8 into ASPC‐1 and Hs766T cells with sh‐GNA15. CCK‐8 and colony formation assays confirmed that CXCL8 could rescue the decreased cell proliferation capacity induced via GNA15 knockdown (Figure 3J–L and Figure S5H). Additionally, Transwell assays demonstrated that the introduction of myc‐CXCL8 restored the impaired invasive and migratory capacities caused by GNA15 silencing (Figure 3M,N and Figure S5I,J). In conclusion, these results suggest that CXCL8 is a downstream target gene of GNA15.

### 2.5 GNA15 Upregulates the Expression of CXCL8 by Activating the Phosphorylation of STAT3

To further investigate the mechanism by which GNA15 regulates CXCL8 expression, we performed combined prediction using multiple databases. Our results indicated that STAT3 and FOS are potential upstream transcription factors of CXCL8 in PDAC (Figure 4A). In addition, mass spectrometry and CO‐IP assays revealed a large number of proteins with potential interaction relationships with GNA15, including STAT3 (Figure 4B,C). Data from TCGA database showed a significantly positive correlation between the mRNA expression levels of STAT3 and CXCL8 in PDAC patients from different clinical cohorts (Figure 4D). Therefore, we hypothesized that GNA15 promotes the transcriptional activation of CXCL8 via STAT3.

**FIGURE 4:**
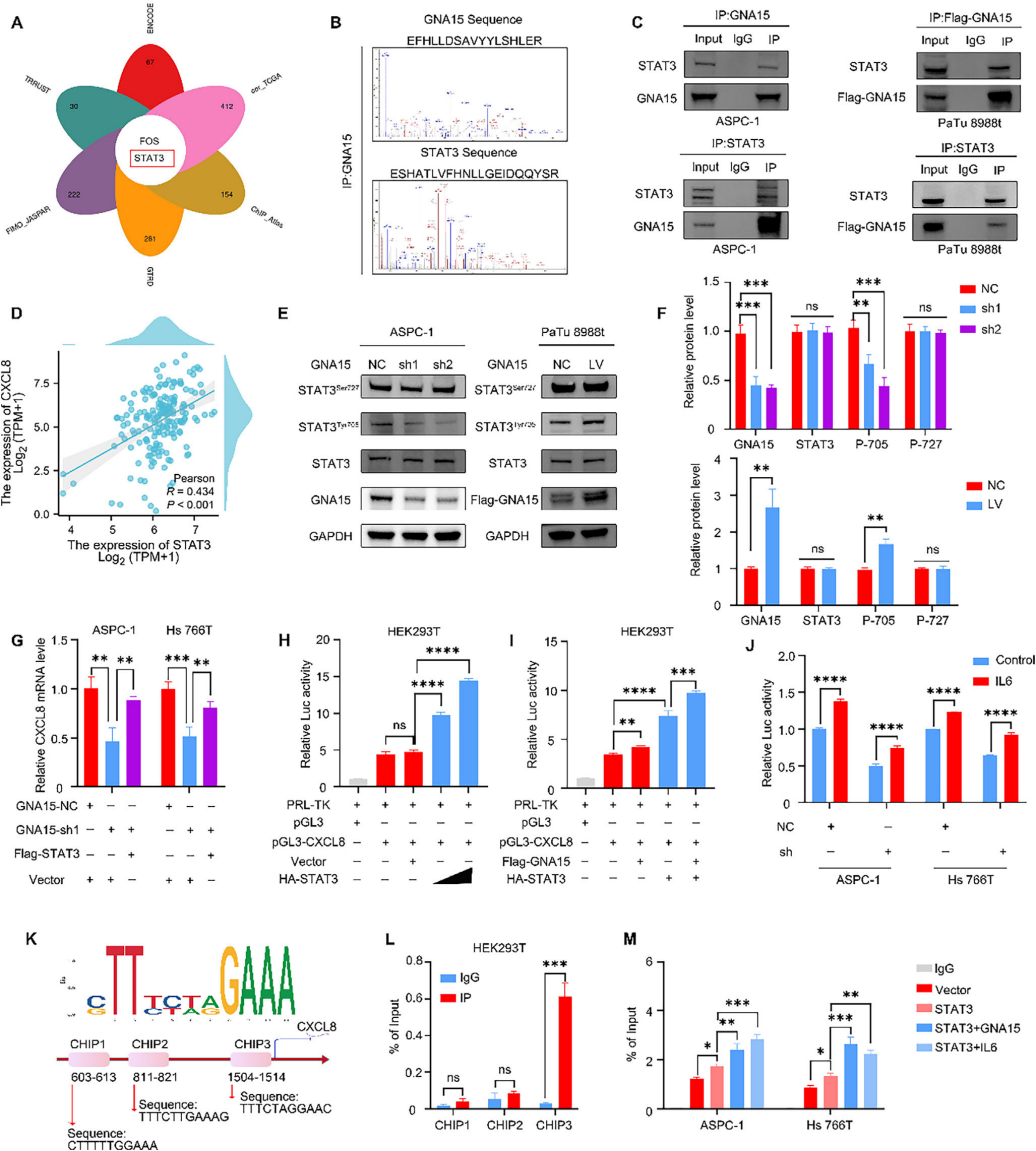
GNA15 upregulates the expression of CXCL8 by activating the phosphorylation of STAT3. (A) Two potential upstream transcription factors of CXCL8 were identified by combined multi‐database analysis. (B) STAT3 was identified as a potential GNA15‐binding protein by IP/MS. (C) Western blot analysis was performed to detect the interaction between GNA15 and STAT3 after co‐immunoprecipitation in PCs. (D) The correlation of expression levels between STAT3 and CXCL8 in the TCGA dataset. (E, F) Western blot analysis was conducted to determine the expression levels of STAT3 and its phosphorylated protein under the condition of GNA15 overexpression or knockdown. (G) The rescue effect of STAT3 overexpression on CXCL8 was detected by qRT‐PCR. (H, I) Dual‐luciferase reporter assay was performed on HEK293T cells with separate overexpression or combined overexpression of STAT3 and GNA15 to measure CXCL8 promoter activity. (J) Dual‐luciferase reporter assay was carried out in PCs stable cell lines with or without IL6 addition to determine CXCL8 promoter activity. (K) Schematic diagram of three potential binding sequences between CXCL8 and STAT3. (L) Chromatin CHIP‐qPCR was used to detect the recruitment level of STAT3 to the CXCL8 promoter in HEK293T cells overexpressing STAT3 and CXCL8. M) CHIP‐qPCR was employed to detect the recruitment level of STAT3 to the CXCL8 promoter in PCs stable cell lines. The *t*‐test was used for comparisons between two groups, and ANOVA was used for multiple group comparisons. *p < 0.05. **p < 0.01. ***p < 0.001. ****p < 0.0001.

To verify this hypothesis, western blot was used to examine the relationship between GNA15 and STAT3. The results demonstrated that GNA15 knockdown significantly inhibited the protein level of STAT3 at the Tyr705 phosphorylation site, whereas no significant changes were observed in the total STAT3 protein level or phosphorylation at the Ser727 site (Figure 4E,F). Extensive previous studies have confirmed that the phosphorylation level of STAT3 at the Tyr705 site directly determines its transcriptional function [28, 29]. Overexpression of STAT3 rescued the downregulation of CXCL8 expression induced by GNA15 knockdown (Figure 4G). Dual‐luciferase reporter assay was used to explore changes in CXCL8 promoter activity, and the results confirmed that GNA15 enhances CXCL8 promoter activity by activating STAT3 phosphorylation (Figure 4H–J). For further verification, we commissioned HZREPOBIO Co., Ltd. to predict the binding region between STAT3 and CXCL8, and designed relevant primers (CHIP1, CHIP2, and CHIP3) targeting CXCL8. Chromatin Immunoprecipitation (ChIP)‐ qPCR analysis showed that STAT3 binds to the CHIP3 region of the CXCL promoter, and activation of STAT3 phosphorylation significantly enhanced the binding of STAT3 protein to the CXCL8 promoter region (Figure 4K–M). Based on these results, we demonstrated that GNA15 upregulates CXCL8 through STAT3.

### 2.6 GNA15 Promotes the Phosphorylation and Dimerization of STAT3

STAT3 is an essential transcription factor that is closely associated with proliferation and drug resistance in pancreatic cancer [30, 31]. Subsequently, we explored how GNA15 activates STAT3 phosphorylation to ultimately upregulate CXCL8 expression. Transcriptomic data analysis revealed no significant alterations in genes associated with the downstream effector pathways (cAMP and PKC pathways) of G proteins (Figure S6A). Therefore, we hypothesized that GNA15 may regulate STAT3 phosphorylation through alternative mechanisms. Previous studies have demonstrated that Gα subunits regulate the phosphorylation level of STAT3 either through the IL‐6 pathway or by direct binding to STAT3 [32, 33]. We aimed to investigate the mechanism by which GNA15 regulates STAT3 phosphorylation at the protein level. Based on prior research, we hypothesized that GNA15 affects STAT3 phosphorylation levels through Janus kinases (JAKs). Firstly, STAT3 dimerization was simulated by transfecting Flag‐STAT3 and HA‐STAT3 plasmids, and CO‐IP assays were performed to verify changes in STAT3 dimerization with GNA15 knockdown or overexpression. The results indicated that overexpression of GNA15 significantly enhanced STAT3 dimerization, whereas knockdown of GNA15 inhibited STAT3 dimerization (Figure 5A,B and Figure S6D,E).

**FIGURE 5:**
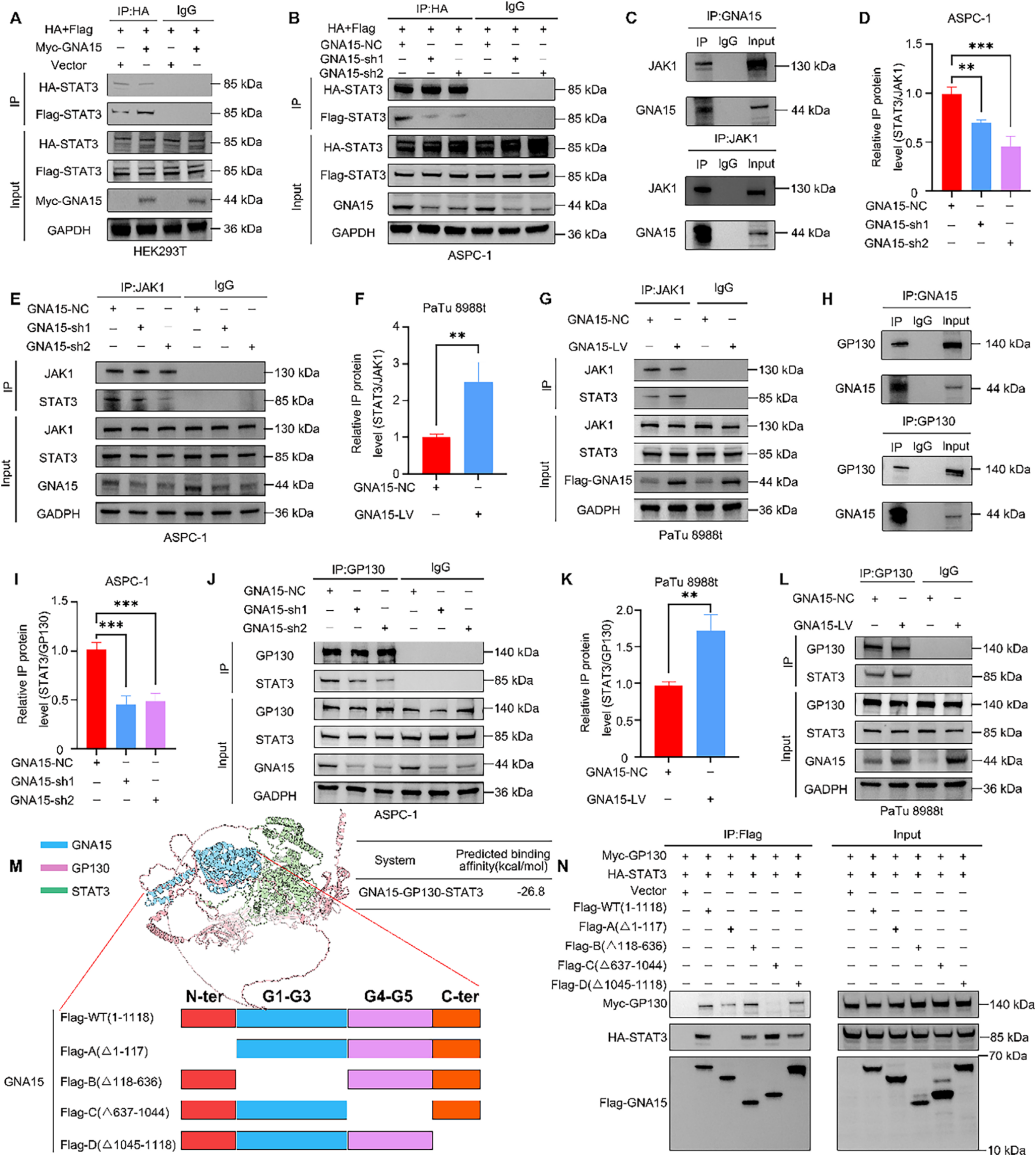
GNA15 promotes the phosphorylation and dimerization of STAT3. (A) HA‐IP assay was performed in HEK293T cells with the indicated plasmids. (B) HA‐IP assay was conducted in ASPC‐1 cells with the indicated plasmids. (C) Western blot analysis was performed to detect the endogenous interaction between GNA15 and JAK1 after co‐immunoprecipitation in ASPC‐1 cells. (D–G) JAK1‐IP assay was carried out in PCs with the indicated conditions. (H) Western blot analysis was performed to detect the endogenous interaction between GNA15 and GP130 after co‐immunoprecipitation in ASPC‐1 cells. (I–L) GP130‐IP assay was conducted in PCs with the indicated conditions. (M, N) Exogenous co‐immunoprecipitation assay of HA‐STAT and Myc‐GP130 with full‐length and mutant forms of Flag‐GNA15 was performed in HEK293T cells. The t‐test was used for comparisons between two groups, and ANOVA was used for multiple group comparisons.

JAK1 is crucial phosphorylating kinases for STAT3 [34]. Mass spectrometry analysis revealed an interaction between GNA15 and JAK1, which was subsequently validated by CO‐IP assays in PCs (Figure 5C and Figure S6B). Interestingly, knockdown of GNA15 in PCs did not affect the protein level of JAK1, but reduced the binding capacity between JAK1 and STAT3 (Figure 5D,E and Figure S6F). In contrast, GNA15 overexpression significantly enhanced the interaction between JAK1 and STAT3 (Figure 5F,G and Figure S6G). GP130, as a key signal transduction protein for JAKs‐mediated STAT3 phosphorylation, can promote the binding of JAKs to STAT3 and enhance STAT3 phosphorylation levels. Mass spectrometry results demonstrated the presence of direct binding peptides between GNA15 and GP130, which was confirmed by CO‐IP assays (Figure 5H and Figure S6C). Additionally, knockdown of GNA15 in PCs significantly inhibited the binding between GP130 and STAT3, whereas the opposite results were observed upon GNA15 overexpression (Figure 5I–L and Figure S6H,I). SC144 [35] and Upadacitinib [36] are small‐molecule inhibitors of GP130 and JAK1, respectively. Following treatment of control and GNA15‐overexpressing PaTu 8988t cells with SC144 and Upadacitinib, we observed an intriguing phenomenon. Both SC144 and Upadacitinib markedly suppressed STAT3 phosphorylation at the Tyr705 site, and such inhibitory effects on STAT3 phosphorylation could not be reversed by GNA15 overexpression. These findings further demonstrate the essential roles of GP130 and JAK1 in GNA15‐mediated activation of STAT3 phosphorylation (Figure S6J,K). Subsequently, molecular dynamics simulations revealed that GNA15 interacts with STAT3 and GP130 through numerous hydrogen bonds and salt bridges, forming a stable ternary complex (Figure S7A‐M). Finally, full‐length GNA15 plasmid (Flag‐WT) and four truncated GNA15 plasmids (Flag‐A, Flag‐B, Flag‐C, and Flag‐D) were transfected into HEK293T cells, followed by CO‐IP assays. We observed that STAT3 interacts with the N‐terminal region of GNA15, while GP130 binds to the G4‐G5 domains of GNA15 (Figure 5M,N). These results suggested that GNA15 promotes the phosphorylation of STAT3 via JAK1. Consequently, we have elucidated the transcriptional mechanism of CXCL8 regulation by GNA15 via STAT3.

### 2.7 GNA15 Promotes M2 Polarization of Macrophages via CXCL8

Previous studies have clearly demonstrated that GNA15 upregulates CXCL8 expression by activating STAT3 phosphorylation. As a secreted protein, the role of CXCL8 regulated by GNA15 in PDAC microenvironment deserves further investigation. To this end, we evaluated the effect of GNA15 knockdown on CXCL8 secretion levels in PCs by enzyme‐linked immunosorbent assay (ELISA). The results indicated that GNA15 knockdown significantly inhibited CXCL8 secretion compared with the control group (Figure 6A). Subsequently, integrated analysis of multiple PDAC‐related single‐cell datasets confirmed that GNA15 and CXCL8 were specifically co‐expressed in macrophages (Figure 6B). Relevant studies have shown that CXCL8 promotes M2 polarization of TAMs via CXCR1/CXCR2, and M2‐type macrophages are closely associated with the malignant phenotype of tumor cells [37]. Therefore, we hypothesized that GNA15 promotes M2 polarization of TAMs through the CXCL8‐CXCR1/CXCR2 axis, and further facilitating the malignant progression of PDAC.​

**FIGURE 6:**
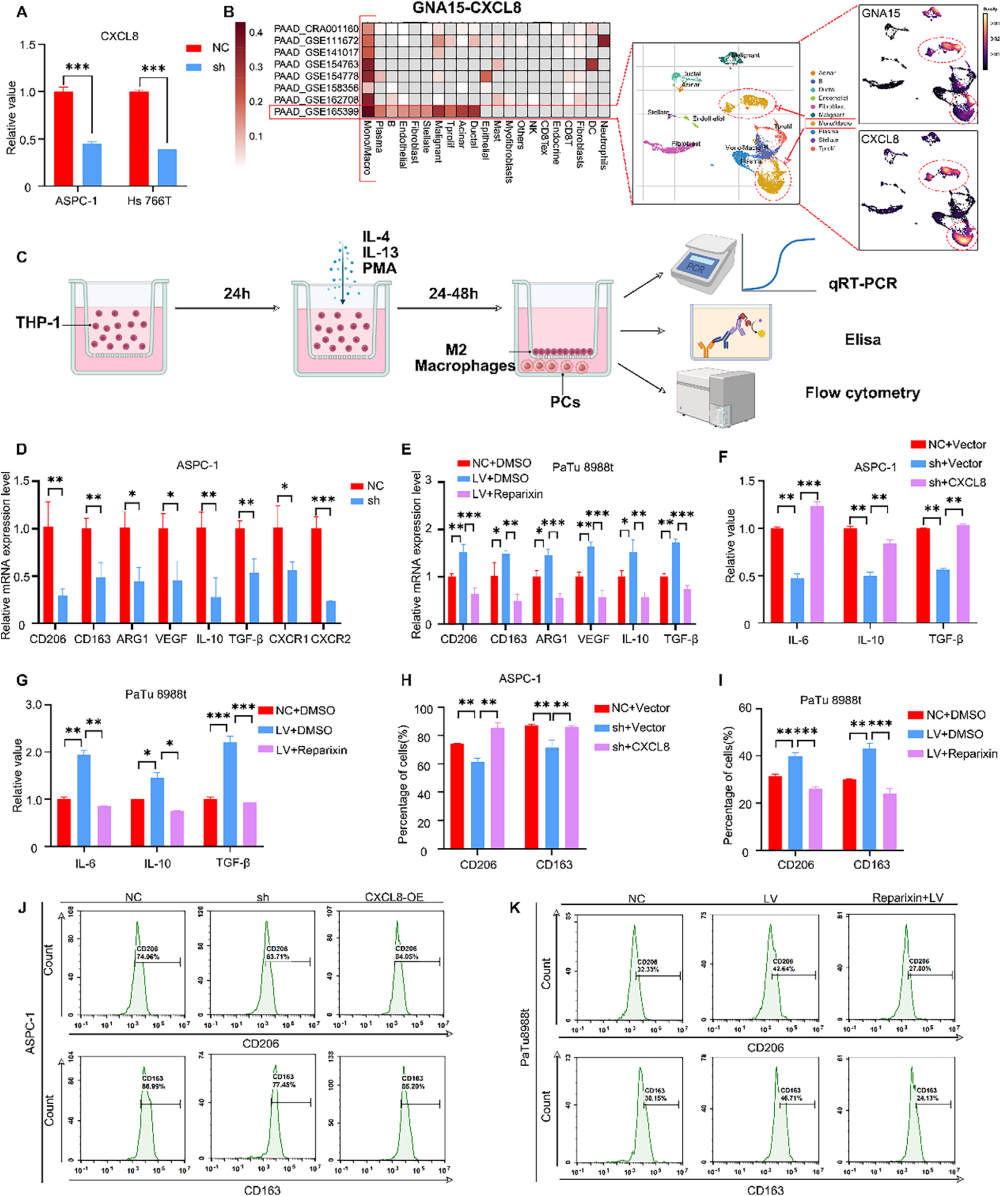
GNA15 promotes M2 polarization of macrophages via CXCL8. (A) ELISA analysis of CXCL8 in PCs. (B) Heatmap of combined analysis of GNA15 and CXCL8 in multiple single‐cell datasets. (C) Schematic diagram of the co‐culture of THP‐1 and PCs. (D, E) qPCR analysis of the relative expression of M2 markers (CD206, CD163, Arginase‐1, VEGF, TGF‐βand IL‐10) in THP‐1‐derived M2 TAMs. (F, G) ELISA analysis of IL‐6, IL10 and TGF‐β in THP‐1‐derived M2 TAMs. (H–K) Percentage of CD206+ and CD163+ cells in THP‐1‐derived M2 TAMs. The t‐test was used for comparisons between two groups, and ANOVA was used for multiple group comparisons. *p < 0.05. **p < 0.01. ***p < 0.001. ****p < 0.0001.

To verify this hypothesis, we established a co‐culture model of THP‐1 cells and PCs in vitro (Figure 6C). RNA level detection showed that the expression levels of CD206, CD163, ARG1, VEGF, IL‐10, TGF‐β, CXCR1, and CXCR2 were downregulated in THP‐1 cells after GNA15 knockdown in PCs (Figure 6D and Figure S8A). In contrast, overexpression of GNA15 in PCs significantly increased the expression levels of CD206, CD163, ARG1, VEGF, IL‐10, and TGF‐β, and this phenomenon could be inhibited by Reparixin (Figure 6E and Figure S8B) [38]. In addition, the results of Elias assay confirmed that the levels of IL‐6, IL‐10, and TGF‐β were decreased in the sh‐GNA15 group, while they were increased in the sh+CXCL8 group (Figure 6F and Figure S8C). The addition of Reparixin to THP‐1 cells could inhibit the upregulation of IL‐6, IL‐10, and TGF‐β induced by GNA15 overexpression (Figure 6G and Figure S8D). Besides, flow cytometry analysis revealed that GNA15 knockdown in PCs reduced the proportions of CD206+ and CD163+ cells, and this phenomenon could be rescued by enhancing CXCL8 expression (Figure 6H,J and Figure S8E,F). On the contrary, increased GNA15 expression in PCs led to an elevation in the proportions of CD206+ and CD163+ cells, which could be reversed by the addition of the Reparixin neutralizing antibody against CXCL8 (Figure 6I,K and Figure S8G–I). To further verify that the promotion of macrophage M2 polarization by GNA15 is dependent on the CXCL8‐CXCR1/CXCR2 axis in vivo, we established a KPC subcutaneous tumor model (Figure 7A). We observed that the tumor volume and weight in the LV‐GNA15 group were significantly increased, whereas Reparixin effectively inhibited tumor growth. Subsequently, TAMs isolated from in vivo PDAC tissues were used for flow cytometry and ELISA detection. The results showed that the percentages of CD206+ and CD163+ cells were increased, the proportion of CD86+ cells was decreased, and the levels of IL‐6, IL‐10, and TGF‐β were elevated in the LV‐GNA15 group. Interestingly, treatment with Reparixin effectively inhibited tumor growth and macrophage M2 polarization (Figure 7B–G). Collectively, these lines of evidence demonstrate that GNA15 in PCs could promote the M2 polarization of TAMs via the CXCL8‐CXCR1/CXCR2 axis.

**FIGURE 7:**
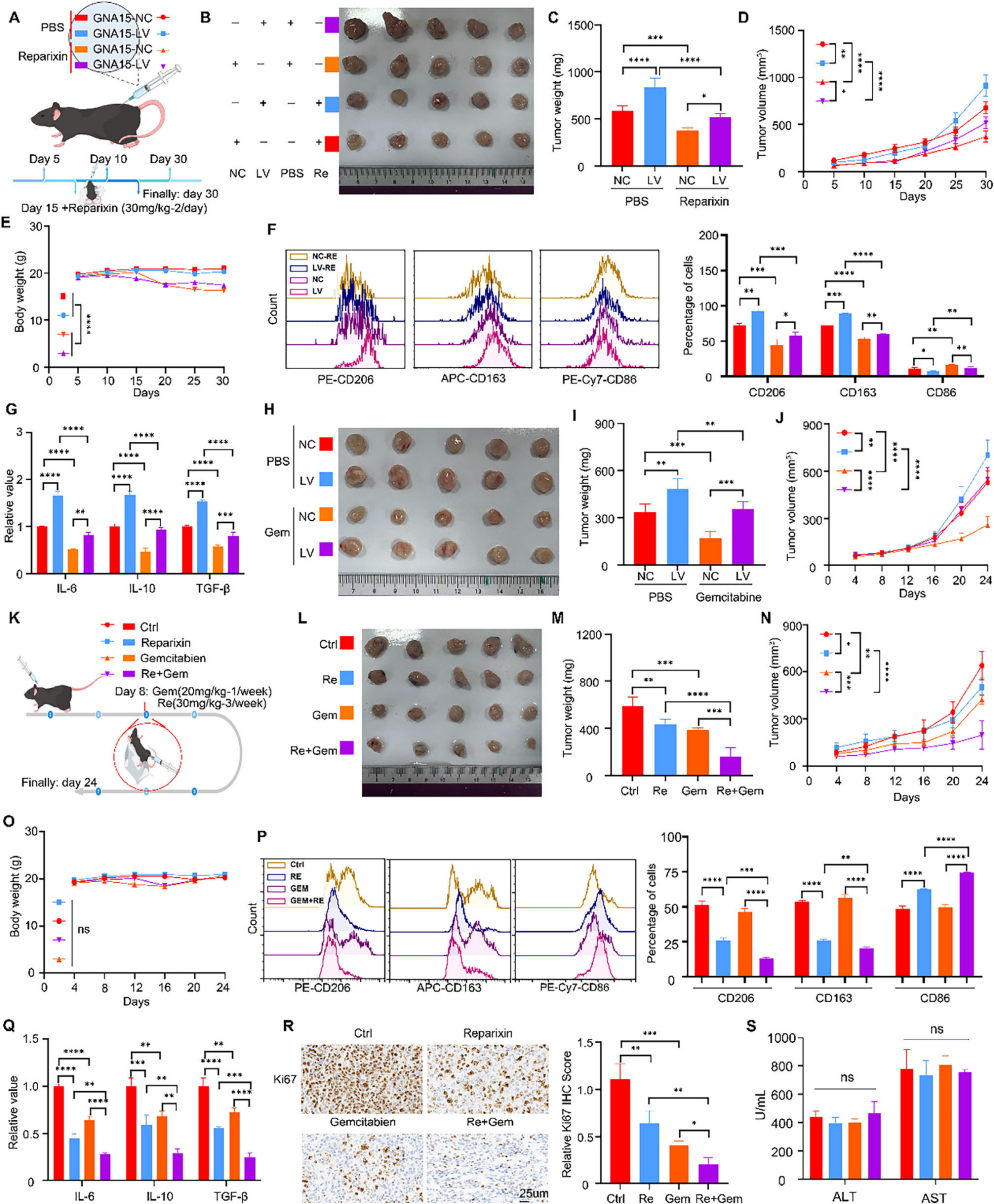
Reparixin Inhibits Gemcitabine Therapeutic Resistance Induced by GNA15. (A, B) KPC cells stably expressing negative control and LV‐GNA15 were implanted into C57BL/6 mice treated with or without Reparixin. (C–E) Tumor weight, tumor volumes, and body weight of KPC‐NC and KPC‐GNA15 allografts treated with or without Reparixin. (F) Flow cytometry analysis of CD206+ macrophages, CD163+ macrophages, and CD86+ macrophages from four groups. (G) ELISA analysis of IL‐6, IL‐10, and TGF‐βfrom four groups. (H–J) KPC cells stably expressing negative control and LV‐GNA15 were implanted into C57BL/6 mice treated with or without Gemcitabine. (K–O) KPC cells were implanted into C57BL/6 mice treated with different therapeutic approaches. (P) Percentage of CD206+ macrophages, CD163+ macrophages, and CD86+ macrophages in TAMs from subcutaneous tumor. (Q) ELISA analysis of IL‐6, IL10 and TGF‐β in TAMs from subcutaneous tumor. (R) IHC score of Ki‐67 were shown in the four groups. (S) ALT and AST analysis in KPC mice. The *t*‐test was used for comparisons between two groups, and ANOVA was used for multiple group comparisons. *p < 0.05. **p < 0.01. ***p < 0.001. ****p < 0.0001.

### 2.8 Reparixin Inhibits Gemcitabine Therapeutic Resistance Induced by GNA15

Currently, Gemcitabine remains the first‐line chemotherapeutic agent for the treatment of PDAC. Considering the role of TAMs in Gemcitabine resistance, we investigated whether abnormally high expression of GNA15 induces Gemcitabine resistance in PDAC [23]. The results from CTRP and GDSC2 datasets showed that high GNA15 expression leads to Gemcitabine resistance (Figure S9A). Through CCK8 and colony formation assays, we observed that GNA15 knockdown increased Gemcitabine therapeutic sensitivity, whereas GNA15 overexpression reduced the sensitivity of PCs to Gemcitabine (Figure S9B–H). Similar results were obtained in the subsequently established KPC subcutaneous tumor model (Figure 7H–J). In addition, we evaluated the efficacy of the combination therapy with Reparixin and Gemcitabine using animal models (Figure 7K). We observed that the Re+Gem significantly inhibited tumor growth (Figure 7L–N). Flow cytometry results demonstrated that combined RE and GEM treatment markedly decreased the proportions of CD206^+^ and CD163^+^ macrophages, while elevating the percentage of CD86^+^ macrophages. Results from and ELISA confirmed that the combination therapy of Re+Gem suppressed the markers of M2 macrophages, while promoting the expression of M1 macrophage markers (Figure 7Q). Additionally, the combination therapy increased the proportion of CD8^+^ T cells and significantly upregulated IFN‐γ production by CD8^+^ T cells (Figure S9I, J). IHC staining results showed that the Re+Gem combination therapy inhibited the proliferation of PCs (Figure 7R). More importantly, the combination therapy of Re+Gem at low concentrations did not exhibit significant drug toxicity (Figure 7O,S and Figure S9K). In summary, abnormally high expression of GNA15 induces Gemcitabine resistance and Re+Gem represents a promising therapeutic approach.

## 3 Discussion

In this study, we combined in vivo and in vitro experiments with clinical tissue samples to thoroughly explore the role and mechanism of GNA15 in the progression of PDAC. We first demonstrated the oncogenic properties and immunosuppressive effect of GNA15 in PDAC. Subsequently, to clarify the oncogenic role of GNA15, we conducted in‐depth investigations into its downstream molecular mechanisms. We found that TET3 promotes GNA15 expression in PDAC through demethylation. We first revealed that, GNA15 directly drives the malignant progression of PDAC by activating the phosphorylation of STAT3 via GP130‐JAK1 signaling, thereby upregulating the expression of CXCL8. Furthermore, in PCs high GNA15 expression enhances the release of CXCL8, promotes the M2 polarization of macrophages, and ultimately further induces tumor immunosuppression (Figure 8).

**FIGURE 8:**
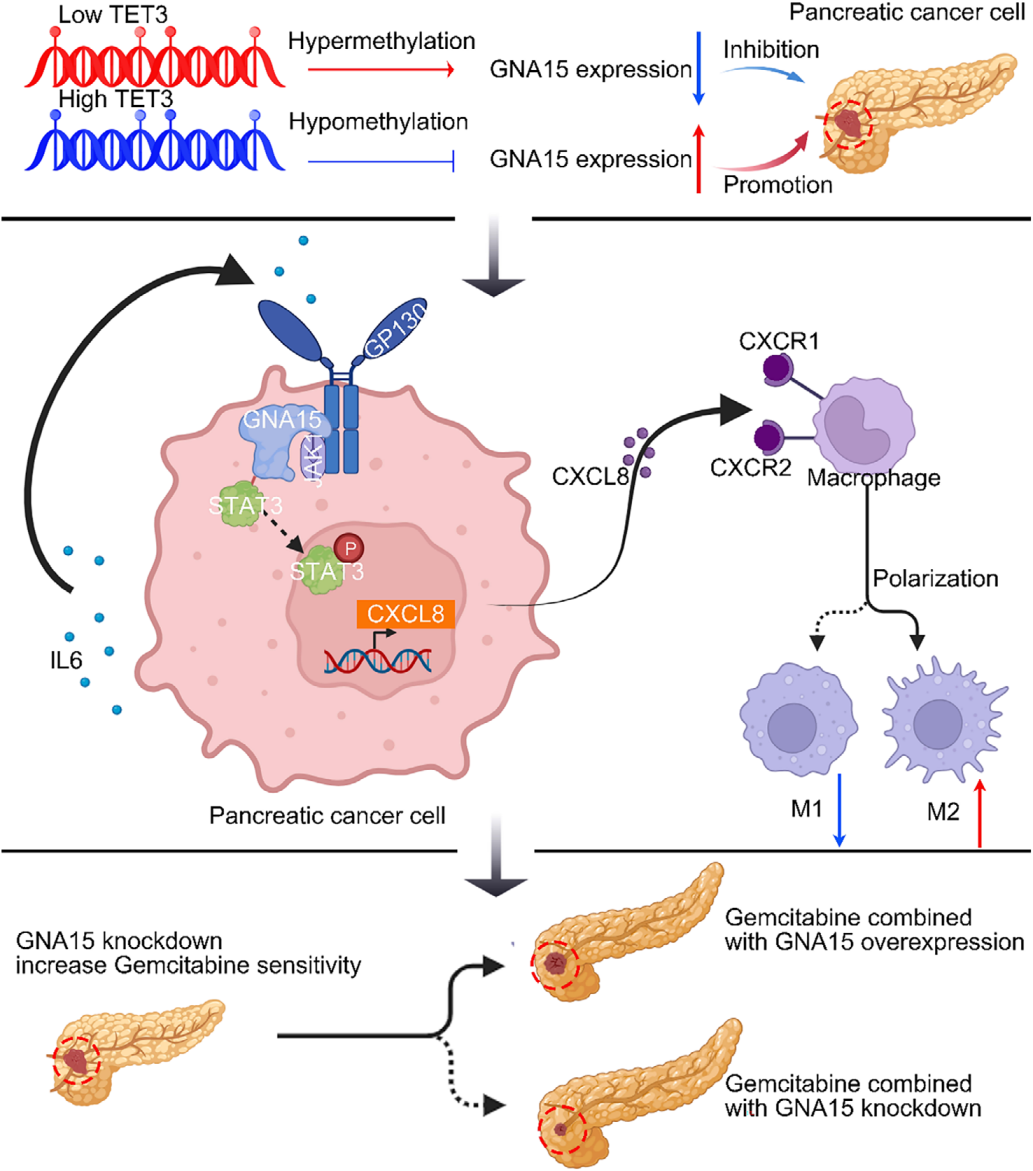
A schematic model summarizing GNA15's mechanism in mediating macrophage M2 polarization and Gemcitabine resistance.

PDAC, as a malignant tumor of the digestive system, has become an arduous medical challenge [39]. GNA15, also known as GNA16 and HG1L, has been demonstrated to play a vital role in tumor proliferation and metastasis [40]. This study demonstrates that GNA15 is highly expressed in PDAC and associated with poor prognosis in patients. Besides, in vitro and in vivo experiments have demonstrated that GNA15 drives the malignant progression of PDAC (Figures 1 and 2). DNA methylation is an important epigenetic modification, which is frequently characterized by dysregulated DNA methylation in tumors. In previous studies, we have revealed the expression relationship between methyltransferases DNMTs and GNA14 [14]. Recent studies have indicated that the abnormally high expression of GNA15 in PDAC is affected by the demethylation of the promoter region [16]. Subsequently, prediction analysis based on the TCGA database showed a significant correlation between TET3 and GNA15. In our study, we confirmed that TET3 is an important demethylase in the demethylation process of GNA15 through TET3 siRNA‐mediated knockdown or Bobcat339 inhibition assays. Furthermore, inhibition of TET3 can partially block GNA15‐driven PDAC progression.

The pathogenesis of PDAC is complex, involving multiple factors such as oncogenes and tumor microenvironment [6, 23]. To further explore the oncogenic mechanism of GNA15, we combined RNA‐seq of NC and sh‐GNA15 with public database information analysis and identified CXCL8 as a downstream gene of GNA15 (Figure 3). Previous studies have indicated that CXCL8, a chemokine, is closely associated with tumor growth, metastasis, and tumor microenvironment [41, 42]. In tumor cells, CXCL8 triggers the activation of the PI3K/Akt signaling pathway, thereby promoting the proliferation and metastasis of tumor cells [43]. To further elucidate the underlying molecular mechanisms, integrated analysis of multiple databases was performed to screen and identify STAT3 as a potential upstream transcription factor of CXCL8. Our study found that high expression of GNA15 upregulates the expression of CXCL8 by activating the phosphorylation of STAT3. Specifically, GNA15 enhances the binding of JAK1 to STAT3 via interacting with GP130 protein, thereby activating STAT3 phosphorylation (Figure 4). The phosphorylated STAT3 translocate into the nucleus to enhance the expression and secretion of CXCL8 (Figure 5). As a member of chemokines, CXCL8 can not only directly promote tumor growth but also regulate the tumor microenvironment in a paracrine manner to affect tumor progression [41]. The tumor microenvironment of PDAC is characterized by massive infiltration of M2‐TAMs [5]. Previous studies have shown that both GNA15 and CXCL8 are associated with M2 polarization of macrophages [37, 44], which is consistent with our findings. In this study, the interaction between GNA15 and the CXCL8‐CXCR1/CXCR2 axis explains the immune suppression in PDAC. In PCs, GNA15 promotes the secretion of CXCL8 by upregulating its expression, and the secreted CXCL8 binds to CXCR1 or CXCR2 on macrophages to promote M2 polarization of macrophages (Figures 6 and 7). These results highlight the important role of the GNA15‐STAT3‐CXCL8 axis in PDAC progression and its potential as a therapeutic target.

Gemcitabine is a first‐line chemotherapeutic agent for the treatment of pancreatic cancer and one of the main modalities for treating this disease [45]. However, Gemcitabine resistance remains a major medical challenge. Emerging evidence has linked TAMs polarization to Gemcitabine resistance, indicating that targeted inhibition of TAMs polarization can effectively enhance the efficacy of Gemcitabine [5]. Our study demonstrates that high expression of GNA15 is associated with Gemcitabine resistance, while combined treatment with low‐dose Reparixin and Gemcitabine exerts effective antiproliferative effects on PDAC via blocking M2 polarization induced by the GNA15‐STAT3‐CXCL8 axis (Figure 7).

In conclusion, we have elucidated a complex regulatory axis involving GNA15, STAT3, and CXCL8 that drives PDAC progression, macrophage polarization, and Gemcitabine resistance. These novel findings suggest that targeting GNA15 is a promising new strategy for inhibiting PDAC progression and enhancing Gemcitabine sensitivity.

## 4 Method

### 4.1 Bioinformatic Analysis

Gene expression data and clinical information for pancreatic cancer patients were obtained from The Cancer Genome Atlas (TCGA). Subsequent analyses were performed using R. For single‐cell RNA sequencing data, Z‐score normalization was applied, with sample similarities assessed based on Euclidean distance matrices. Ward's hierarchical clustering was used to optimize the arrangement of rows and columns, and the results were visualized in a heatmap. For spatial transcriptomics data, deconvolution analysis was used to reclassify tissue regions: spots containing any malignant cells (>0%) were defined as malignant (Mal), while those without malignant cells (0%) were classified as non‐malignant (nMal). Differential gene expression between these two groups (Mal and nMal) was evaluated using the Wilcoxon test in R. Finally, average gene expression levels for each group were presented using bar plots.

### 4.2 Clinical Specimens

Tissue samples, including tumor and peritumoral tissues, were obtained from 30 patients with PDAC who received treatment at Zhejiang Provincial People's Hospital from June 2020 to June 2025, and all samples were stored in liquid nitrogen. These samples were used for RT‐qPCR and Western blot analyses. All tissue samples were confirmed to be PDAC by the Department of Pathology, Zhejiang Provincial People's Hospital. Informed consent was provided by all patients prior to surgery, and this study was approved by the Ethics Committee of Zhejiang Provincial People's Hospital (QT2025266).

### 4.3 Cell Culture

KPC, PaTu 8988t, Hs 766T, MIA PaCa‐2 and HPDE were cultured in Dulbecco's Modified Eagle Medium (DMEM) supplemented with 10% FBS. ASPC‐1 was cultured in RPMI 1640 medium supplemented with 10% FBS.

### 4.4 Transfection and Lentivirus Infection

The plasmids and small interfering RNAs (siRNAs) used in this study were purchased from HZREPOBIO.

The GNA15 knockdown and overexpression lentiviruses used in this study were purchased from Genechem.

Lipomaster 3000 transfection agent (TL301‐01/02, Vazyme) was used to transfection according to the manufacturer's instructions. GNA15‐overexpression and short hairpin RNA (shRNA) knockdown lentivirus were used to infect PDAC cell lines, and puromycin (G418, Thermo Fisher Scientific) was used to screen stable PDAC cell lines.

### 4.5 Western Blot Analysis and CO‐IP

Cells from 10‐cm diameter culture dishes were harvested, washed twice with phosphate‐buffered saline (PBS), and lysed for 30 min using a cell lysis buffer supplemented with phosphatase inhibitors (P0013, Beyotime). Total cellular protein was then extracted for Western blot and immunoprecipitation assays. The protein concentration was determined using a bicinchoninic acid (BCA, E112‐01/02, Vazyme) assay, followed by immunoprecipitation (IP) with rProtrinAG (rProteinAG, SM015005, Smart‐Lifesciences/ Flag‐016‐101‐003/ HA‐003‐101‐003, alpvhhs) as per the manufacturer's instructions. Gels for electrophoresis were prepared with a PAGE Gel Kit (E304‐01, Vazyme).

### 4.6 Quantitative Real‐Time PCR Analysis

RNA was extracted following the manufacturer's instructions (R112, Vazyme). Subsequently, reverse transcription and quantitative PCR were performed using designated kits (R333 for reverse transcription and Q711 for quantitative PCR, Vazyme).

### 4.7 Cell Proliferation Assay

To assess the proliferative capacity of pancreatic cancer cells, we conducted both CCK‐8 and colony formation assays.

CCK‐8 assay: 2,500 cells were plated per well in 96‐well plates. Cell proliferation was evaluated every 24 h using the Cell Counting Kit‐8 (CCK‐8, abs50003, absin) according to the manufacturer's instructions.

Colony formation assay: Cells were seeded in 6‐well plates at densities ranging from 500 to 2000 cells per well. After being cultured for 2–3 weeks, the colonies were fixed and stained with 1% crystal violet, followed by manual counting.

### 4.8 Transwell Assay

The migratory and invasive capacities of pancreatic cancer cells were assessed using a Transwell assay.

We seeded 5 × 10^4^ cells in 200 µL serum‐free medium into the upper chambers of Transwell plates, which were either uncoated or pre‐coated with Matrigel (GL101‐01, Vazyme) to evaluate migration and invasion. The lower chambers contained 700 µL of medium with 20% FBS as a chemoattractant. After 24 or 48 h, the cells that had traversed the membrane were fixed, stained with 1% crystal violet, and manually counted.

### 4.9 Flow Cytometry

Cell cycle and apoptosis were analyzed using the Cell Cycle Assay Kit (E‐CK‐A353, Elabscience) and the Apoptosis Assay Kit (A213‐01, Vazyme), respectively, according to the manufacturers' protocols. For immunophenotyping, harvested cells were washed twice with PBS, resuspended in 100 µL of staining buffer, which contained 5 µL of the corresponding antibody, and incubated for 30 min. Finally, the stained cells were subjected to flow cytometry.

### 4.10 In Vivo Tumor Model

We obtained the relevant animal ethics from the Animal Center of Zhejiang Provincial People's Hospital (20250225553661).

In the subcutaneous xenograft model, mice were inoculated subcutaneously with LV‐GNA15 or sh‐GNA15 transfected MIA PaCa‐2, KPC, or ASPC‐1 cells, as well as negative control cells. Once the xenografts reached a volume of 100 mm^3^, the mice were given intraperitoneal injections of Gemcitabine (60 mg/kg, MCE) every four days. Tumor volumes were monitored throughout the treatment period.

For the lung colonization model, sh‐GNA15 ASPC‐1 cells and negative control cells were injected via the tail vein.

### 4.11 Dual‐Luciferase Reporter Assay

To assess promoter activity, HEK‐293T cells were seeded in 6‐well plates at 200,000 cells per well and cultured for 24 h prior to co‐transfection with a CXCL8 promoter construct and STAT3/GNA15 plasmids. Luciferase activity was measured using the Dual‐Luciferase Reporter Assay Kit (DL101‐01, Vazyme) according to the manufacturer's instructions.

### 4.12 Chromatin Immunoprecipitation (ChIP)

For the CHIP (Chromatin Immunoprecipitation) assay, we meticulously performed all relevant procedures in strict accordance with the standardized protocols provided by the manufacturer (Rk 20258, ABclonal).

### 4.13 Elisa

Coculture 200,000 THP‐1‐derived M2 macrophages with PCs that either overexpress or do not overexpress GNA15 in a 6‐well plate for 48 h. After incubation, collect the culture supernatants and measure the concentrations of IL‐10, IL‐6, and TGF‐β by using the human IL‐10 ELISA kit (catalog number SEKH‐0018, Solarbio), the human IL‐6 ELISA kit (catalog number SEKH‐0014, Solarbio), and the human TGF‐β ELISA kit (catalog number SEKH‐0316, Solarbio), respectively.

### 4.14 Protein–Protein Docking

In the present study, we employed the AlphaFold Server to predict the binding modes of protein trimers. Firstly, we obtained the sequence files of these proteins separately from the Uniprot Database, which were then submitted to the AlphaFold Server for fully automated prediction. Upon completion of the prediction, the top‐ranked conformation (Top1) was subjected to binding energy evaluation using the online tool PRODIGY (https://wenmr.science.uu.nl/prodigy/). The binding mode with the optimal binding energy was further analyzed for visualization using PyMOL 2.5.3. The binding affinity of the protein trimers was derived from the docking scores. Negative values of binding affinity indicate the potential for binding interaction, and the more negative the value, the stronger the binding affinity.

### 4.15 Statistical Analysis

The t‐test was used for comparisons between two groups, and analysis of variance (ANOVA) was used for multiple group comparisons. P<0.05 was labeled as statistically significant. *p < 0.05. **p < 0.01. ***p < 0.001. ****p < 0.0001.

## Author Contributions

**Zhen‐Yuan Qian**: conceptualization, supervision, funding acquisition, resources, project administration. **Lei Wang**: formal analysis, investigation. **Ji Xu**: funding acquisition, project administration, supervision, resources, conceptualization. **Wei‐Lang Xu**: investigation, formal analysis. **Kai Jiang**: funding acquisition. **Yu‐Xin Weng**: investigation, formal analysis. **Zai‐Yuan Ye**: project administration, supervision, resources. **Guang‐Yuan Song**: data curation, writing – original draft, conceptualization, writing – review and editing, methodology, project administration. **Wei‐Hui Guo**: data curation, formal analysis, investigation, writing – original draft, validation, visualization, writing – review and editing, software.

## Conflicts of Interest

The authors declare that they have no known competing financial interests or personal relationships that could have appeared to influence the work reported in this paper.

## Supporting information




**Supporting File**: advs76860‐sup‐0001‐SuppMat.docx

## Data Availability

Data will be made available on request.
