# Supplementary material for: Hypomethylation of GNA15 Promotes Pancreatic Ductal Adenocarcinoma Progression and Macrophage M2 Polarization via STAT3‐CXCL8 Axis
Source: Adv Sci (Weinh). 2026 Jul 30:e76860. Online ahead of print. doi: 10.1002/advs.76860 (PMC13423485; doi:10.1002/advs.76860)
Supplement: Supplementary file 1 — Supporting File: advs76860‐sup‐0001‐SuppMat.docx [file ADVS-9999-e76860-s001.docx]

# **Supplementary information**

## **Supplementary Figures and Figure legends**

**Figure S1:**


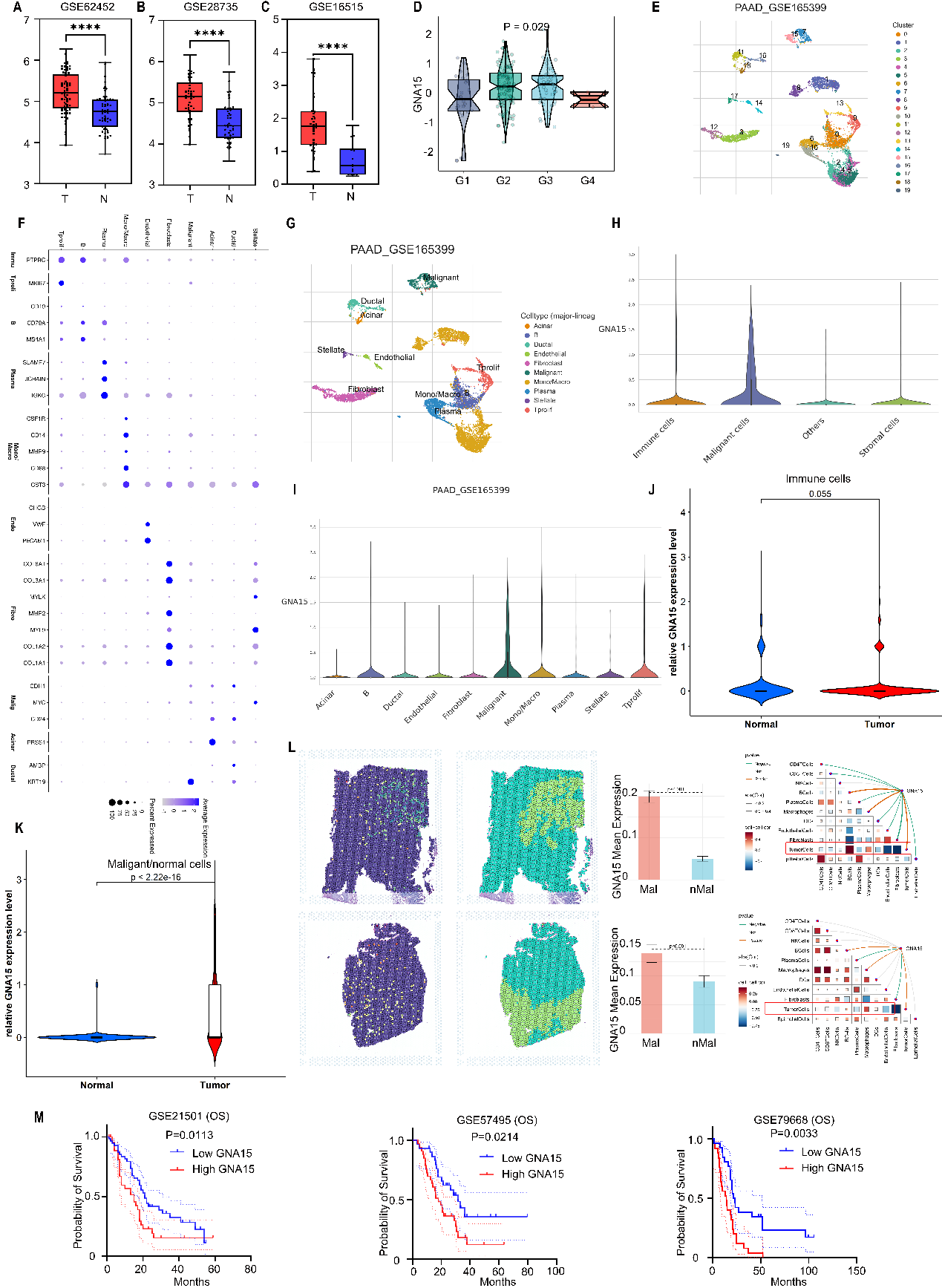


A-C) Expression levels of GNA15 mRNA in PDAC tissues and normal controls from GEO database. D) The association of GNA15 mRNA levels with grade of PDAC patients in the TCGA database. E-K) Analysis of GNA15 in the PDAC Single-cell transcriptome from GSE165399 dataset. L) Analysis of GNA15 in the PDAC spatial transcriptome from GSE211895 dataset. M) The association of GNA15 mRNA levels with OS of PDAC patients in the GEO database.

**Figure S2:**


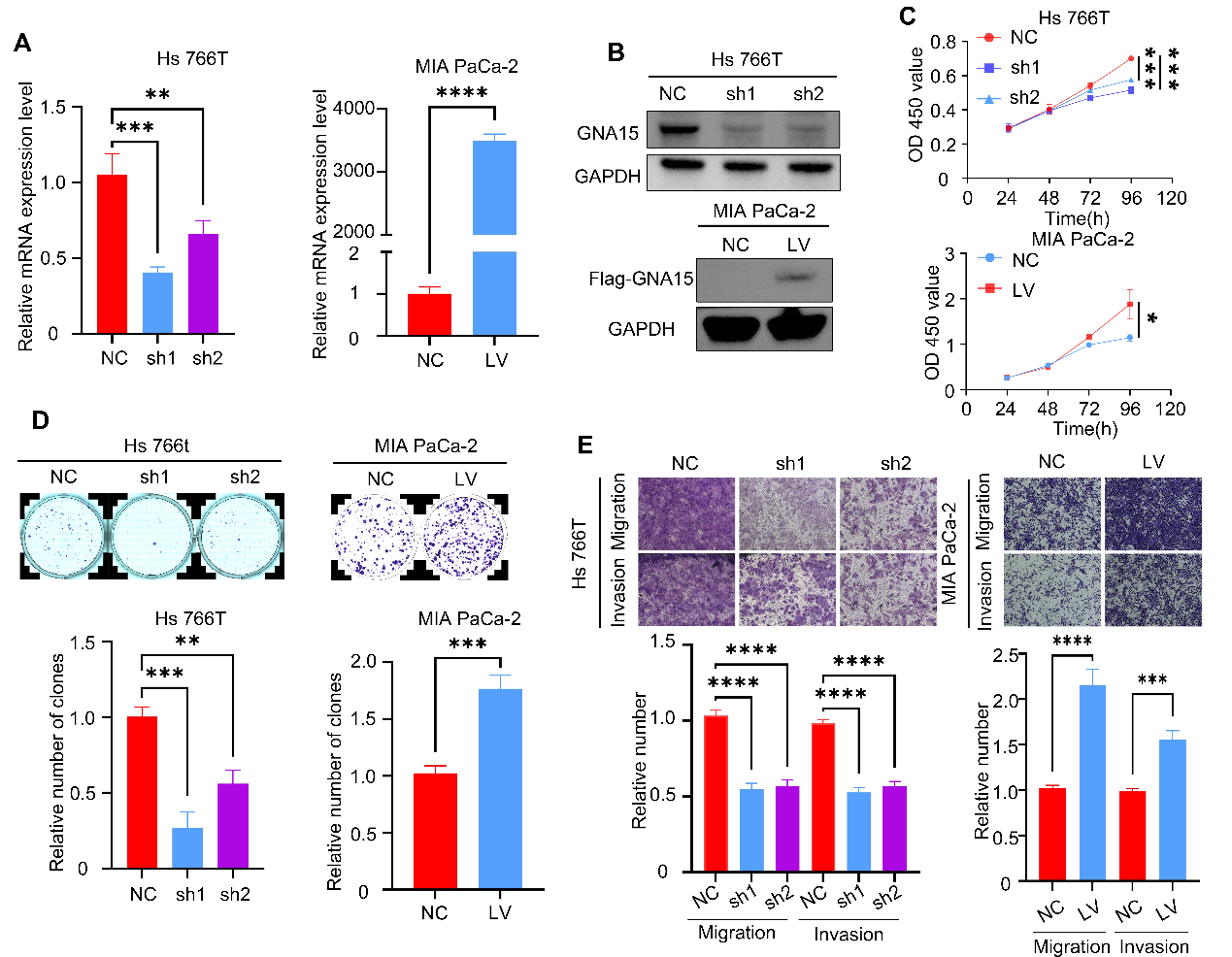


A, B) Verification of lentivirus-mediated stably sh-GNA15 Hs 766T cells and stably LV-GNA15 MIA PaCa-2 cells by qRT-PCR and Western blot. C-E) Hs 766T and MIA PaCa-2 cells were subjected to colony formation assay, CCK-8 assay and Transwell assay.

**Figure S3:**


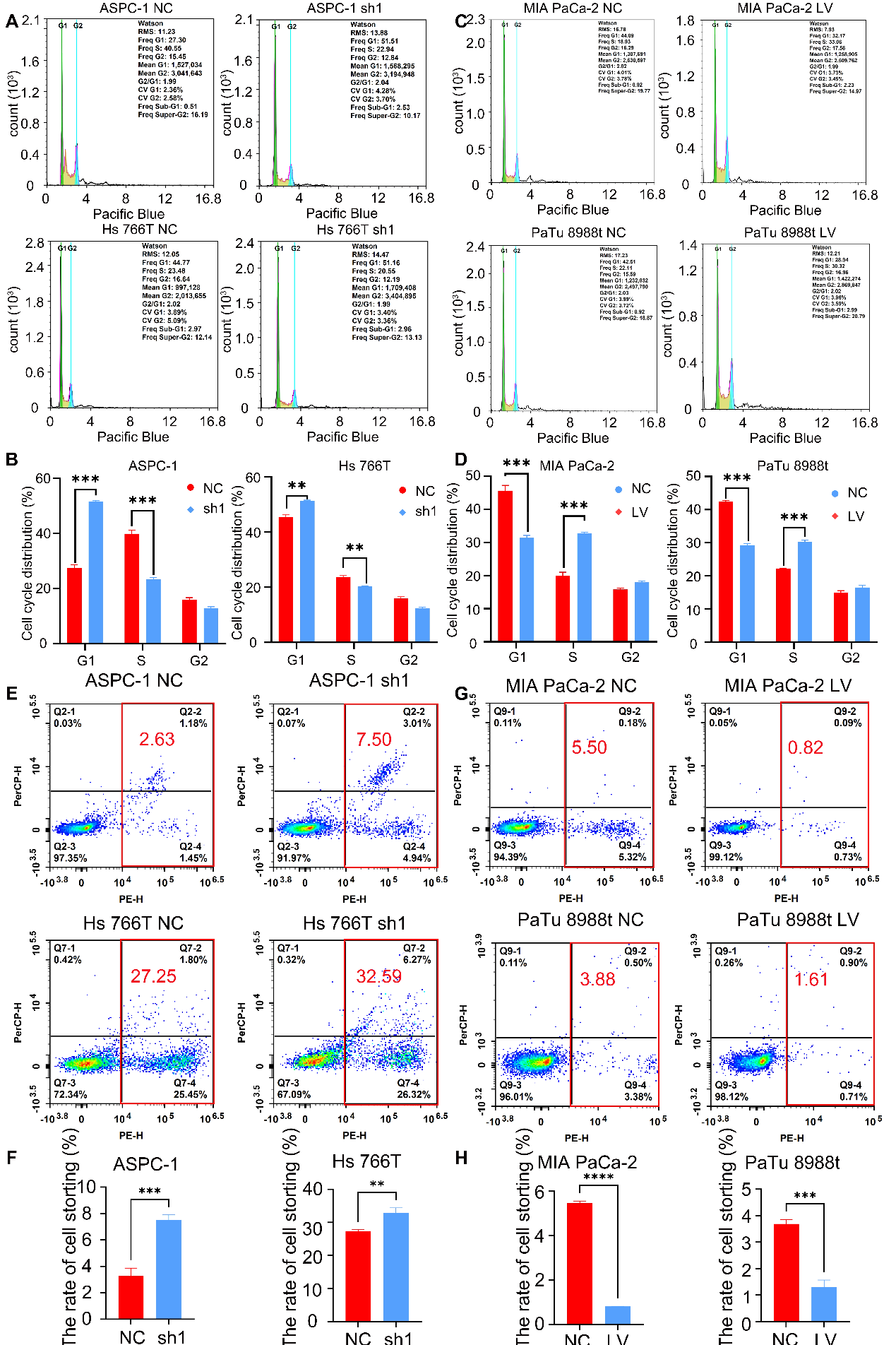


A-D) Flow cytometry was used to analyze the cell cycle changes of stable PCs cell lines. E-H) Flow cytometry was employed to analyze the apoptotic changes of stable PCs cell lines.

**Figure S4:**


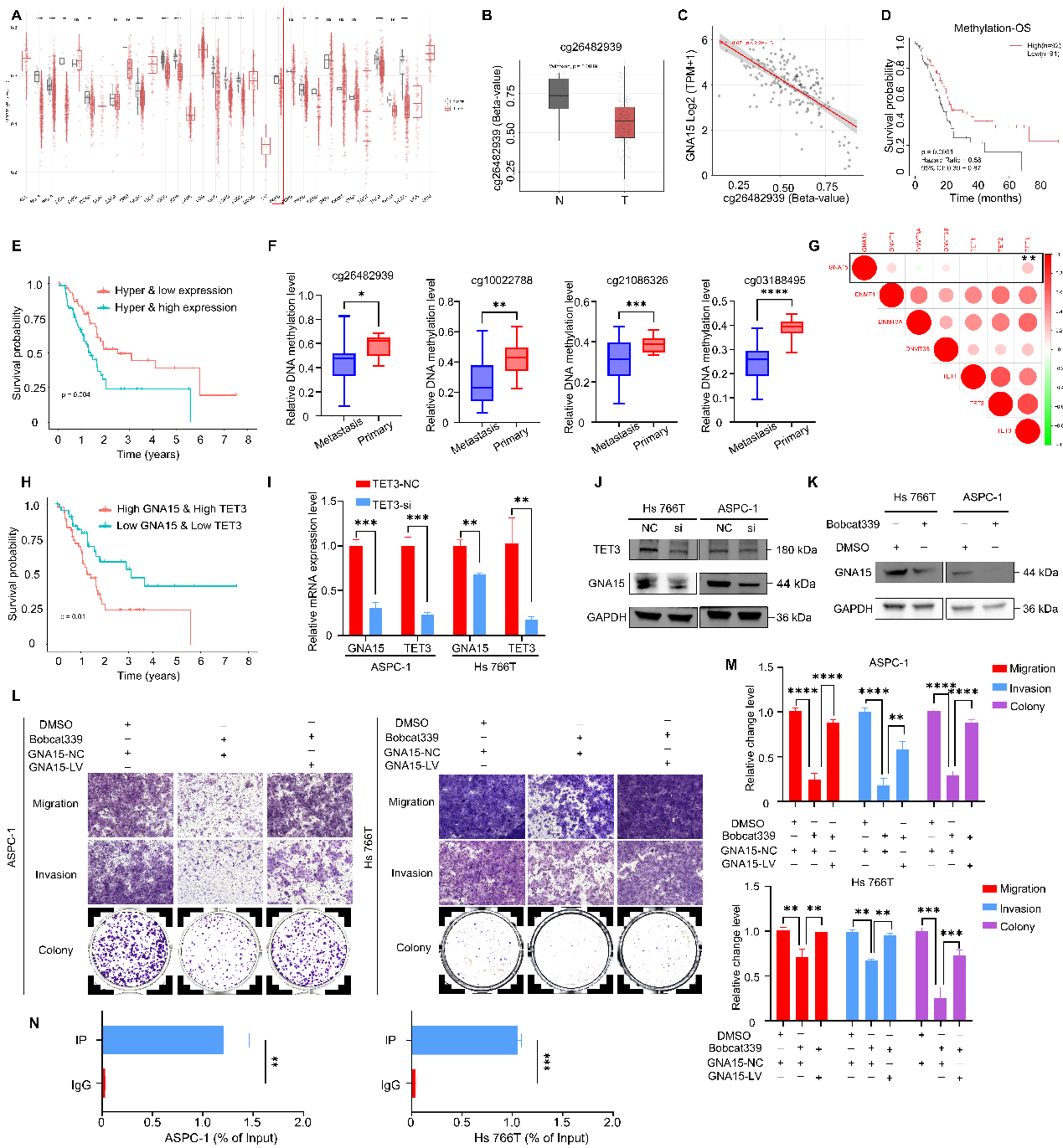


A-D) Correlation analysis of GNA in PDAC using the SMART database. E) R studio was used to perform combined prognostic analysis of GNA15-related methylation and mRNA expression levels in the TCGA database. F) Correlation analysis between methylation levels of different GNA15 methylation sites and PDAC metastasis in the GSE217384 dataset. G) Correlation of expression levels between different DNA methylation-related enzymes and GNA15 in the TCGA database. H) R studio was used to conduct combined prognostic analysis of mRNA expression levels of GNA15 and TET3 in the TCGA database. I, J) Western blot and qRT-PCR were used to detect the expression level of GNA15 after TET3 knockdown in PCs. K) Western blot was employed to detect the protein expression level of GNA15 after TET3 inhibition by Bobcat339. L, M) Functional experiments were performed to detect the effect of Bobcat339 on PCs cells. N) CHIP-qPCR was employed to detect the recruitment level of TET3 to the GNA15 promoter in PCs cell lines.

**Figure S5:**


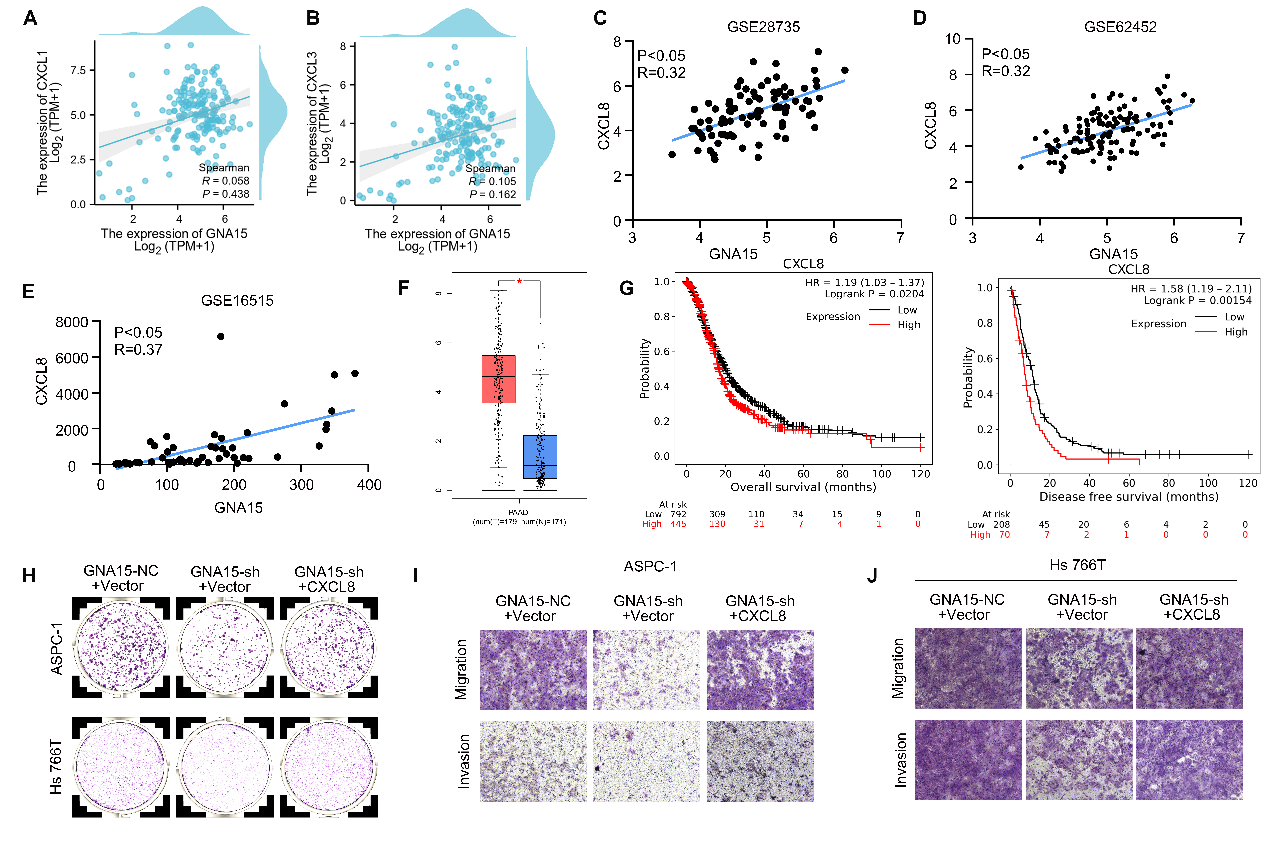


A-E) The association of the mRNA expression levels of GNA15 with CXCL1/CXCL3 in TCGA and GEO databases. F) Expression levels of CXCL8 mRNA in PDAC tissues and normal controls from TCGA database. G) The association of CXCL8 mRNA levels with OS and RFS of PDAC patients in the Kaplan–Meier plotter dataset. H-J) PCs were subjected to colony formation Transwell assay.

**Figure S6:**


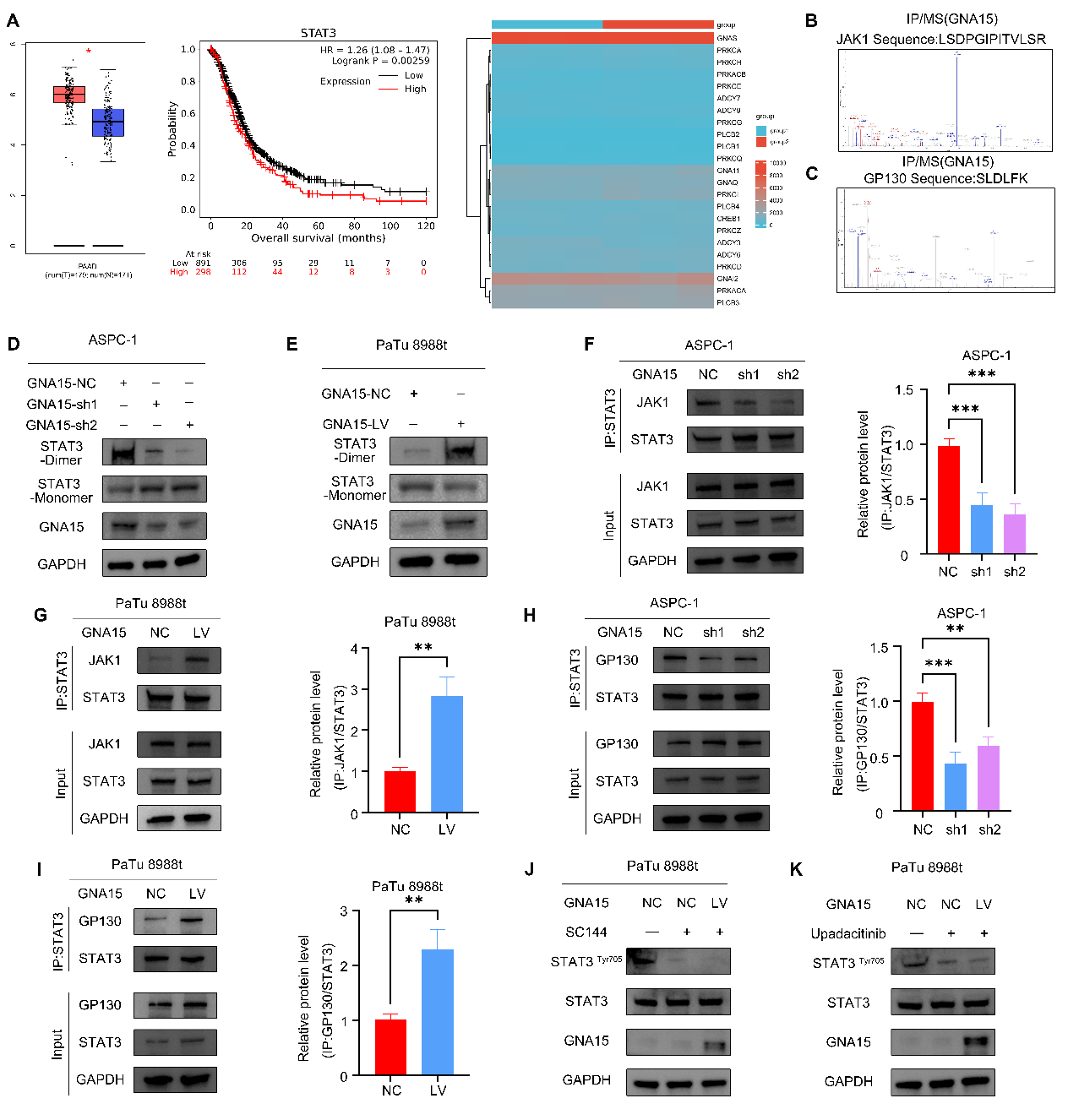


A) Expression levels of STAT3 mRNA in PDAC tissues and normal controls from TCGA database. The association of STAT3mRNA levels with OS of PDAC patients in the Kaplan–Meier plotter dataset. Heatmap of altered genes associated with the cAMP and PKC signaling pathways in RNA-seq analysis. B, C) JAK1 and GP130 were identified as potential GNA15-binding proteins by IP/MS. D, E) Non-reducing immunoblotting was performed to detect the dimerization level of STAT3. F-I) GNA15-IP assay was carried out in PCs with the indicated plasmids. J, K) Activation status of STAT3 under different treatment conditions.

**Figure S7：**


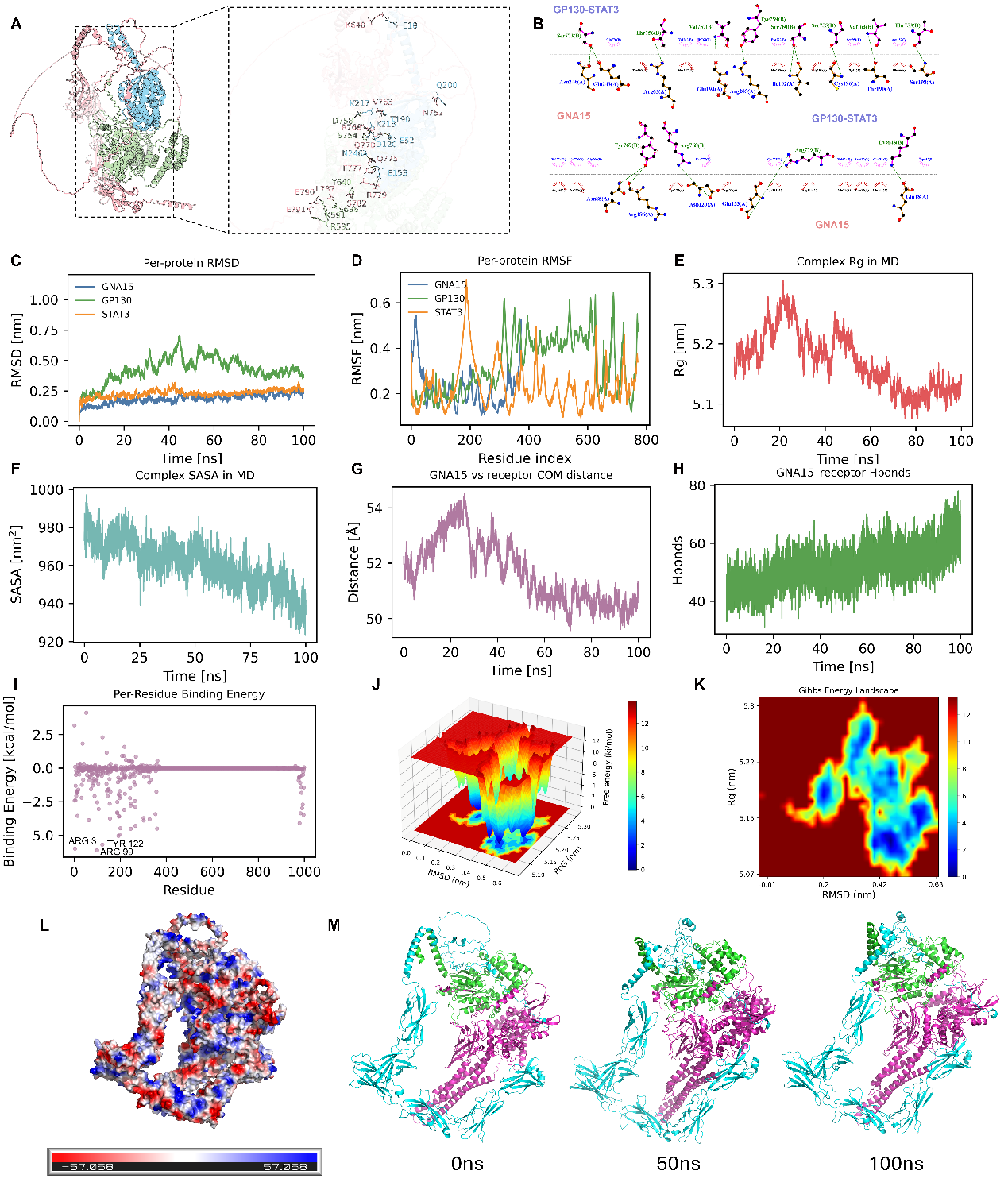


A) The binding mode of the GNA15-GP130-STAT3 complex. The right panel shows a magnified view of the binding site, illustrating the interaction patterns between key residues. Yellow dashed lines represent hydrogen bonds, and purple dashed lines indicate salt bridges. B) Two-dimensional schematic diagram of protein-protein interactions. C) Root-mean-square deviation (RMSD) changes of proteins and the complex during the 100 ns molecular dynamics simulation. D) Root-mean-square fluctuation (RMSF) distribution of protein backbone residues. E) Radius of gyration (Rg) changes of the complex throughout the simulation. F) Time-dependent changes in the solvent-accessible surface area (SASA) of the complex. G) Dynamic variations in the distance between the centroid of the ligand and the protein centroid. H) Fluctuations in the number of protein-protein hydrogen bonds during the simulation process. I) Energy contribution analysis of key amino acid residues to the binding free energy. J) Three-dimensional free energy landscape (FEL) constructed based on RMSD and Rg values. K) Two-dimensional projection of the free energy landscape. L) Electrostatic potential distribution on the surface of the protein-protein complex. M) Conformational comparison of the complex at 0 ns, 50 ns, and 100 ns during molecular dynamics simulation.

**Figure S8:**


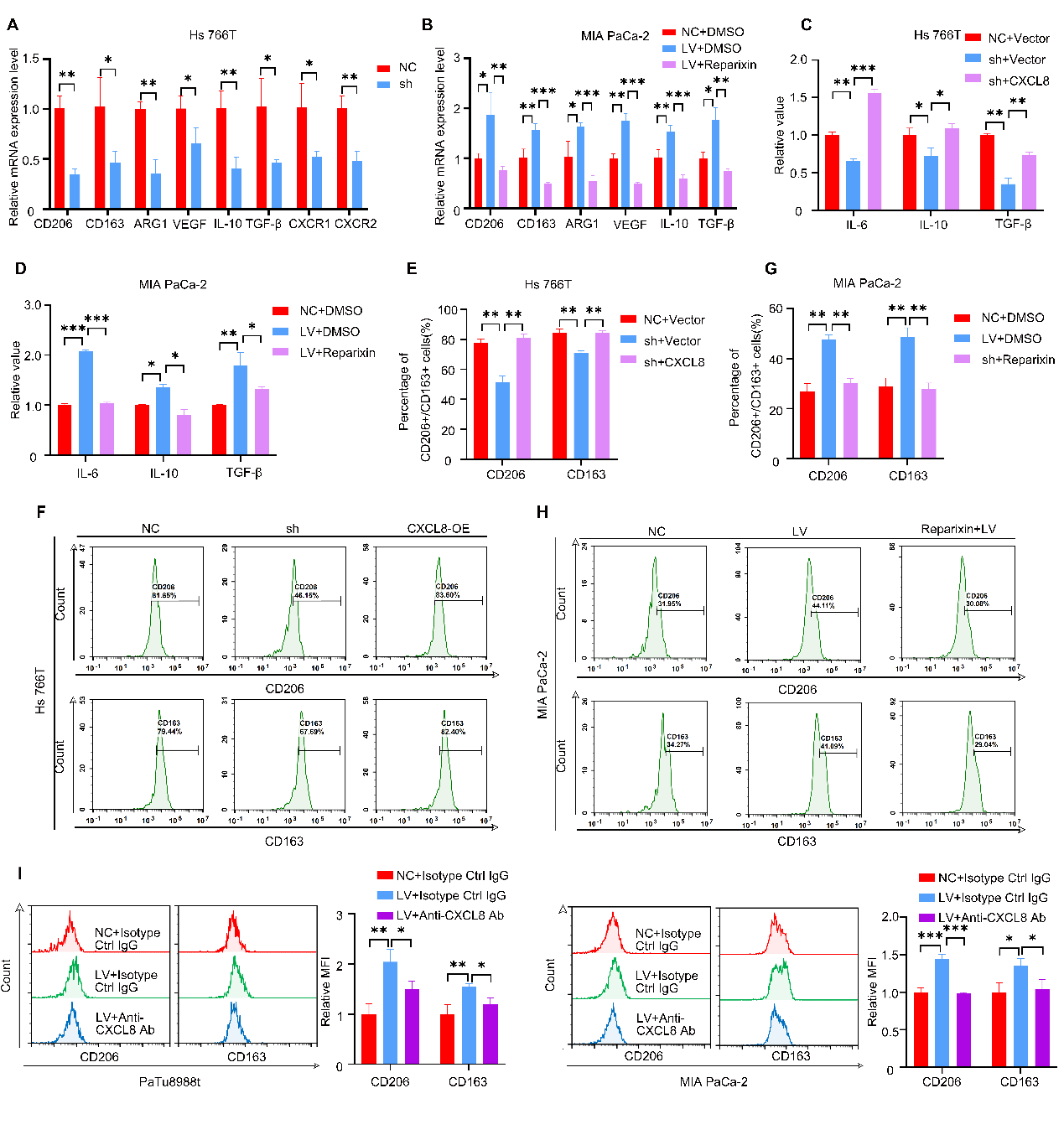


A, B) qPCR analysis of the relative expression of M2 markers (CD206, CD163, Arginase-1, VEGF, TGF-βand IL-10) in THP-1-derived M2 TAMs. C, D) ELISA analysis of IL-6, IL10 and TGF-β in THP-1-derived M2 TAMs. E-H) Percentage of CD206+ and CD163+ cells in THP-1-derived M2 TAMs. I) Mean fluorescence intensity of CD206 versus CD163 in THP-1-derived M2-type TAMs.

**Figure S9:**


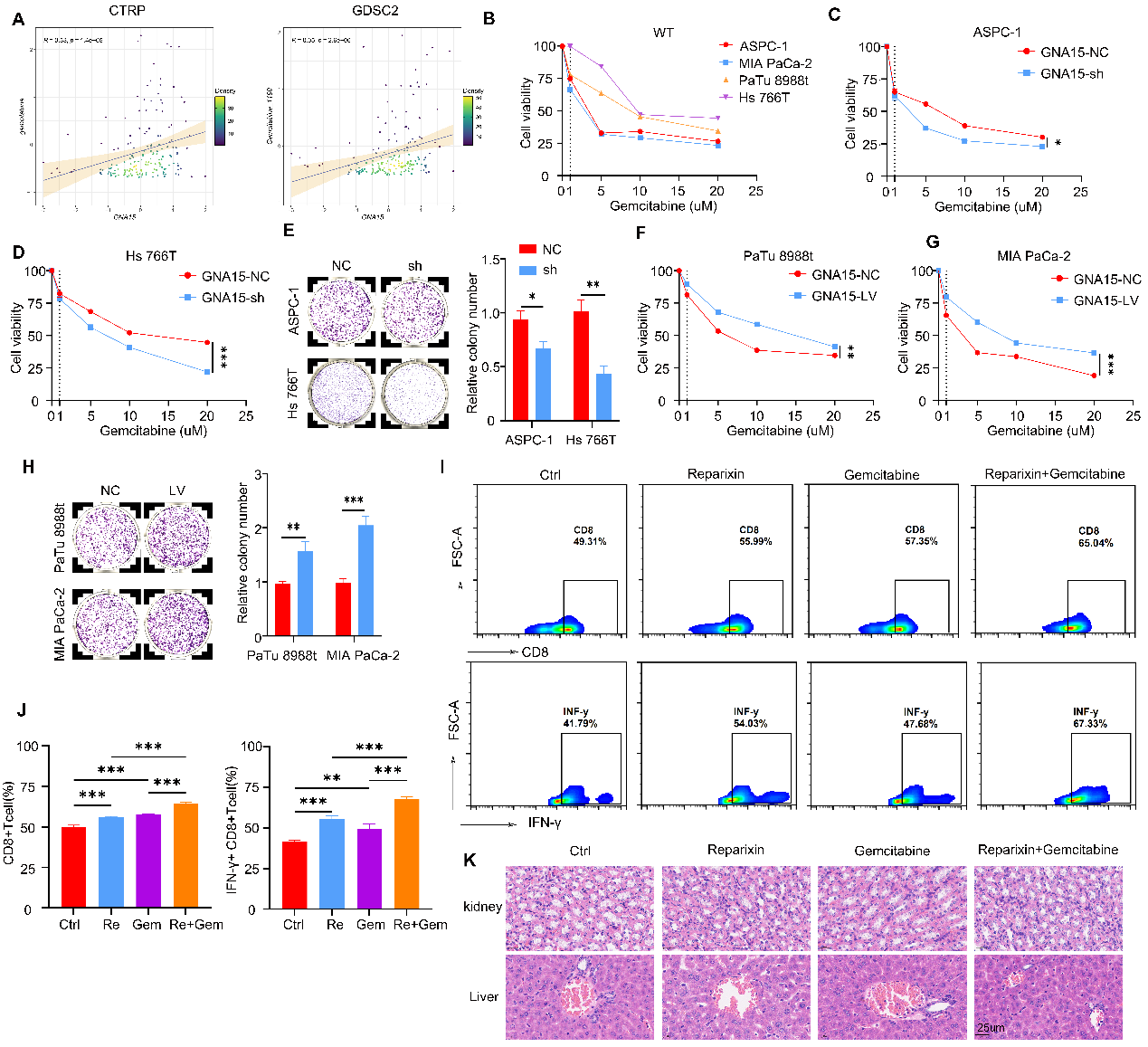


A) Analysis of GNA15 mRNA expression levels and Gemcitabine drug sensitivity from the CTRP and GDSC2 databases. B) Analysis of cell viability with different doses of gemcitabine in wild-type PCs cells. C-H) Detection of cell viability and proliferation ability of PCs cells after GNA15 knockdown or overexpression. I, J) Proportion of CD8+T cells and IFN-γ+CD8+T cells in subcutaneous tumors. K) HE staining of the kidneys and livers from mice treated with different therapies.

**Supplementary Tables Supplementary**

**Supplementary Table S1. The primers used for qRT-PCR**

| **Gene** | **Sequence** |
| --- | --- |
| **H-CXCL1-F** | **GCTTGCCTCAATCCTGCAT** |
| **H-CXCL1-R** | **TCCTCCTCCCTTCTGGTCAG** |
| **H-CXCL3-F** | **CCAAACCGAAGTCATAGCCAC** |
| **H-CXCL3-R** | **TGCTCCCCTTGTTCAGTATCT** |
| **H-CXCL8-F** | **TCTGCAGCTCTGTGTGAAGG** |
| **H-CXCL8-R** | **TGGGGTGGAAAGGTTTGGAG** |
| **H-STAT3-F** | **ACCAGCAGTATAGCCGCTTC** |
| **H-STAT3-R** | **GCCACAATCCGGGCAATCT** |
| **H-CXCR1-F** | **CTGACCCAGAAGCGTCACTT** |
| **H-CXCR1-F** | **ATGGTAAGCCTGGCGGAAAA** |
| **H-CXCR2-F** | **GTTTCGCCATGGACTCCTCA** |
| **H-CXCR2-R** | **AGTGTGCCCTGAAGAAGAGC** |
| **H-CD206-F** | **AGGATGGGTACTGGGCAGAT** |
| **H-CD206-R** | **CTGGACCTTGGCTTCGTGAT** |
| **H-CD163-F** | **AGGCATGACTCACAACGATTCT** |
| **H-CD163-R** | **CCAGGTGTGGCTCTGCATTT** |
| **H-ARG1-F** | **GTCTGTGGGAAAAGCAAGCG** |
| **H-ARG1-R** | **AATGTAGCTGTGGTCTCCGC** |
| **H-IL10-F** | **CCTGACCACGCTTTCTAGCT** |
| **H-IL10-R** | **GGCTCCCTGGTTTCTCTTCC** |
| **H-CXCL8-CHIP-F1** | **AGAATTCAGTGTAACCCAGGCA** |
| **H-CXCL8-CHIP-R1** | **TCACCTTGGTGTAACAGTTCTTCA** |
| **H-CXCL8-CHIP-F2** | **TACTTGCCCAGAAGCGAACA** |
| **H-CXCL8-CHIP-R2** | **AGTCTGACCTCTGAGGACCC** |
| **H-CXCL8-CHIP-F3** | **TCCCCCACATTACTCAGAAAGT** |
| **H-CXCL8-CHIP-R3** | **TGGTGAAGATAAGCCAGCCA** |
| **H-GAPDH_2_F (H, M, R)** | **GAACGGGAAGCTCACTGG** |
| **H-GAPDH_2_R (H, M, R)** | **GCCTGCTTCACCACCTTCT** |
| **H-TET3-F** | **TCCAGCAACTCCTAGAACTGAG** |
| **H-TET3-R** | **AGGCCGCTTGAATACTGACTG** |
| **H-GNA15-F** | **GATCCATTGTTTCGAGAACGTGA** |
| **H-GNA15-R** | **GGTAGTTCCAGGATAGTCCCAA** |
| **H-TGF-F** | **TACCTGAACCCGTGTTGCTCTC** |
| **H-TGF-R** | **GTTGCTGAGGTATCGCCAGGAA** |
| **H-VEGF-F** | **TTGCCTTGCTGCTCTACCTCCA** |
| **H-VEGF-R** | **GATGGCAGTAGCTGCGCTGATA** |

**Supplementary Table S2. Chemical reagents used in this study**

| **Chemical reagents** | **Vendors** | **Cat#** |
| --- | --- | --- |
| **Bobcat339** | **MCE** | **HY-111558A** |
| **Reparixin** | **MCE** | **HY-15251** |
| **Gemcitabine** | **MCE** | **HY-17026** |

**Supplementary Table S3. shRNA and siRNA sequences**

| **Gene** | **sequence** |
| --- | --- |
| **shGNA15** | **1: CAGGATCCTCTTGGAGCAGAA**  **2: TGGCATCAACGAGTACTGCTT**  **3: ATGGATCCATTGTTTCGAGAA** |
| **si1TET3** | **F: GCCUGUGGUUCCUCCUGAATT**  **R: UUCAGGAGGAACCACAGGCTT** |
| **si2TET3** | **F: GACCCUUACAGCAUGAACATT**  **R: UGUUCAUGCUGUAAGGGUCTT** |
| **si3TET3** | **F: GGCUCUAUGAAACCUUCAATT**  **R: UUGAAGGUUUCAUAGAGCCTT** |

**Supplementary Table S4. Antibodies used in this study**

| **Antibodies** | **Vendors** | **Cat#** |
| --- | --- | --- |
| **GNA15** | **Proteintech** | **12078-1-AP** |
| **GNA15** | **Bioss** | **Bs-13248R** |
| **STAT3** | **Proteintech** | **10253-2-AP** |
| **STAT3-Tyr705** | **Cell Signaling Technology** | **9145** |
| **STAT3-Ser727** | **Cell Signaling Technology** | **34911** |
| **JAK1** | **Proteintech** | **66466-1-Ig** |
| **GP130** | **Proteintech** | **67766-1-Ig** |
| **TET3** | **Proteintech** | **27150-1-AP** |
| **CXCL8** | **Abclonal** | **A24736** |
| **CD206** | **Abclonal** | **A28215** |
| **CD163** | **Abclonal** | **A27963** |
| **CD86** | **Abclonal** | **A27409** |
| **CD206 (H)** | **Abclonal** | **A27493** |
| **CD163 (H)** | **Abclonal** | **A27960** |

**Supplementary Table S5. Clinicopathologic characteristics of 120 PDAC patients from Zhejiang Provincial People Hospital in tissue microarrays**

| Parameters |  | case |
| --- | --- | --- |
| Sex | MALE | 68 |
|  | FEMALE | 52 |
| Age | <60 | 30 |
|  | ≥60 | 90 |
| Diameter(cm) | <5 | 103 |
|  | ≥5 | 17 |
| Lymph node metastasis | Yes | 74 |
|  | No | 46 |
| Distant metastasis | Yes | 7 |
|  | No | 113 |
| Vascular invasion | Yes | 42 |
|  | No | 78 |
| Perineuronal invasion | Yes | 91 |
|  | No | 29 |
